# Supplementary material for: Antibacterial Methyl Ester Cembranoids from the Soft Coral Sarcophyton ehrenbergi and Their Structural Elucidation
Source: Mar Drugs. 2025 Apr 15;23(4):170. doi: 10.3390/md23040170 (PMC12028930; doi:10.3390/md23040170)
Supplement: Supplementary file 1 [file marinedrugs-23-00170-s001.zip › marinedrugs-3569575-supplementary.pdf]

Antibacterial methyl ester cembranoids from the soft coral *Sarcophyton ehrenbergi* and their structural elucidation

Meng-Jun Wu[1,†], Song-Wei Li[2,†], Fei Xu[3], Ming-Zhi Su[4], Yue-Wei Guo[2,4,*]

[1] Liangzhu Laboratory, Zhejiang University Medical Center, 1369 West Wenyi Road, Hangzhou 311121, China mow@zju.edu.cn (M.-J.W.); fxu23@zju.edu.cn (F.X.);

[2] School of Medicine, Shanghai University, Shanghai 200444, China; songweili@shu.edu.cn (S.-W.L.);

[3] Department of Gastroenterology of the Second Affiliated Hospital and Institute of Pharmaceutical Bio-technology, School of Medicine, Zhejiang University, Hangzhou 310058, China; fxu23@zju.edu.cn (F.X.);

[4] Shandong Laboratory of Yantai Drug Discovery, Bohai Rim Advanced Research Institute for Drug Discovery, Yantai 264117, China.

*Correspondence: ywguo@simm.ac.cn (Y.-W.G.)

[†] These authors contributed equally to this work.

Content

Table S1. MIC values (μg/mL) of compounds **1–9** and positive controls against ten human pathogens. 4
Table S2. MIC values (μg/mL) of Compounds **1–9** and positive controls against eight sea fish pathogens. 4
1. Original spectra of 1....5
Figure S1. The ^{1}H NMR spectrum of **1**....5
Figure S2. The ^{13}C NMR spectrum of **1**....5
Figure S3. The HSQC spectrum of **1**. 6
Figure S4. The HMBC spectrum of **1**. 6
Figure S5. The ^{1}H–^{1}H COSY spectrum of **1**. 7
Figure S6. The NOESY spectrum of **1**....7
Figure S7. The HR-ESIMS spectrum of **1**....8
Figure S8. The IR spectrum of **1**....8
Figure S9. The ECD and UV spectra of **1**....8
2. Original spectra of 2....9
Figure S10. The ^{1}H NMR spectrum of **2**....9
Figure S11. The ^{13}C NMR spectrum of **2**....9
Figure S12. The HSQC spectrum of **2**. 10
Figure S13. The HMBC spectrum of **2**. 10
Figure S14. The ^{1}H–^{1}H COSY spectrum of **2**. 11
Figure S15. The NOESY spectrum of **2**....11
Figure S16. The HR-ESIMS spectrum of **2**....12
Figure S17. The IR spectrum of **2**....12
Figure S18. The ECD and UV spectra of **2**....12
3. Original spectra of 3....13
Figure S19. The ^{1}H NMR spectrum of **3**....13
Figure S20. The ^{13}C NMR spectrum of **3**....13
Figure S21. The HSQC spectrum of **3**. 14
Figure S22. The HMBC spectrum of **3**. 14
Figure S23. The ^{1}H–^{1}H COSY spectrum of **3**. 15
Figure S24. The NOESY spectrum of **3**....15
Figure S25. The HR-ESIMS spectrum of **3**....16
Figure S26. The IR spectrum of **3**....16
4. Original spectra of 4....17
Figure S27. The ^{1}H NMR spectrum of **4**....17
Figure S28. The ^{13}C NMR spectrum of **4**....17
Figure S29. The HSQC spectrum of **4**. 18
Figure S30. The HMBC spectrum of **4**. 18
Figure S31. The ^{1}H–^{1}H COSY spectrum of **4**. 19
Figure S32. The NOESY spectrum of **4**....19
Figure S33. The HR-ESIMS spectrum of **4**....20

Figure S34. The IR spectrum of **4**. 20
Figure S35. The ECD and UV spectra of **4**. 20
5. Original spectra of 5....21
Figure S36. The ^{1}H NMR spectrum of **5**. 21
Figure S37. The ^{13}C NMR spectrum of **5**. 21
Figure S38. The HSQC spectrum of **5**. 22
Figure S39. The HMBC spectrum of **5**. 22
Figure S40. The ^{1}H–^{1}H COSY spectrum of **5**. 23
Figure S41. The NOESY spectrum of **5**. 23
Figure S42. The HR-ESIMS spectrum of **5**. 24
Figure S43. The IR spectrum of **5**. 24
Figure S44. The ECD and UV spectra of **5**. 24
6. Original spectra of 6....25
Figure S45. The ^{1}H NMR spectrum of **6**. 25
Figure S46. The ^{13}C NMR spectrum of **6**. 25
Figure S47. The HSQC spectrum of **6**. 26
Figure S48. The HMBC spectrum of **6**. 26
Figure S49. The ^{1}H–^{1}H COSY spectrum of **6**. 27
Figure S50. The NOESY spectrum of **6**. 27
Figure S51. The HR-ESIMS spectrum of **6**. 28
Figure S52. The IR spectrum of **6**. 28
Figure S53. The ECD and UV spectra of **6**. 28
7. Original spectra of 7....29
Figure S54. The ^{1}H NMR spectrum of **7**. 29
Figure S55. The ^{13}C NMR spectrum of **7**. 29
8. Original spectra of 8....30
Figure S56. The ^{1}H NMR spectrum of **8**. 30
Figure S57. The ^{13}C NMR spectrum of **8**. 30
9. Original spectra of 9....31
Figure S58. The ^{1}H NMR spectrum of **9**. 31
Figure S59. The ^{13}C NMR spectrum of **9**. 31
10. Acetylation reaction of 5....32
Figure S60. NMR spectra comparation of the acetylated derivative of **5** (**5a**) and **6**. 32

Table S1. MIC values (μg/mL) of compounds **1–9** and positive controls against ten human pathogens.

Compound	*Staphylococcus aureus* ATCC27154	*Enterococcus faecium*	*Escherichia coli* ATCC25922	*Enterobacter cloacae* ZR042	*Enterobacter hormaechei* 2R043	*pseudomonas aeruginosa* ATCC10145	*Pseudomonas aeruginosa* 2200	*Escherichia coli*	MRSA	*Candida albicans* ATCC 76485
**1**	>39.0	>39.0	>39.0	>39.0	>39.0	>39.0	>39.0	>39.0	>39.0	>39.0
**2**	>38.8	>38.8	>38.8	>38.8	>38.8	>38.8	>38.8	>38.8	>38.8	>38.8
**3**	>33.0	>33.3	>33.5	>33.9	>33.10	>33.11	>33.13	>33.14	>33.16	>33.17
**4**	>34.6	>34.6	>34.6	>34.6	>34.6	>34.6	>34.6	>34.6	>34.6	>34.6
**5**	>33.2	>33.2	>33.2	>33.2	>33.2	>33.2	>33.2	>33.2	>33.2	>33.2
**6**	>39.7	>39.7	>39.7	>39.7	>39.7	>39.7	>39.7	>39.7	>39.7	>39.7
**7**	>33.2	>33.2	>33.2	>33.2	>33.2	>33.2	>33.2	>33.2	>33.2	>33.2
**8**	>37.4	>37.4	>37.4	>37.4	>37.4	>37.4	>37.4	>37.4	>37.4	>37.4
**9**	>31.6	>31.6	>31.6	>31.6	>31.6	>31.6	>31.6	>31.6	>31.6	>31.6
Tetracycline	0.750204	>24.045								
Oxytetracycline	<0.193	>24.844								
levofloxacin	<0.155	9.945								
Ampicillin	<0.145	18.57								

Table S2. MIC values (μg/mL) of Compounds **1–9** and positive controls against eight sea fish pathogens.

Compound	*Streptococcus parauberis* KSP28	*Lactococcus garvieae* MP5245	*Streptococcus parauberis* SPOF3K	*Aeromonas salmonicida* AS42	*Phoyobacterium damselae* FP2244	*Pseudomonas fulva* ZXM181	*Photobacterium halotolerans* LMG 22194T	*Enterobacter cloacae*
**1**	>39.0	>39.0	>39.0	>39.0	>39.0	>39.0	>39.0	>39.0
**2**	38.8	>38.8	>38.8	>38.8	>38.8	>38.8	>38.8	>38.8
**3**	>33.1	>33.2	>33.4	>33.6	>33.7	>33.8	>33.12	>33.15
**4**	>34.6	>34.6	>34.6	>34.6	>34.6	>34.6	>34.6	>34.6
**5**	>33.2	>33.2	>33.2	>33.2	>33.2	>33.2	>33.2	>33.2
**6**	>39.7	>39.7	>39.7	>39.7	>39.7	>39.7	>39.7	>39.7
**7**	>33.2	>33.2	>33.2	>33.2	>33.2	>33.2	>33.2	>33.2
**8**	37.4	>37.4	>37.4	>37.4	>37.4	>37.4	>37.4	>37.4
**9**	31.6	>31.6	>31.6	>31.6	>31.6	>31.6	>31.6	>31.6
Tetracycline	[illegible].05625	0.750204	>24.045	6.1125	0.750204			
Oxytetracycline	1.552	<0.38	12.422	0.387	<0.193			
levofloxacin	1.24	4.97	1.243	<0.155	<0.155			
Ampicillin	4.642	0.29	0.579	>18.57	<0.145			

1. Original spectra of 1

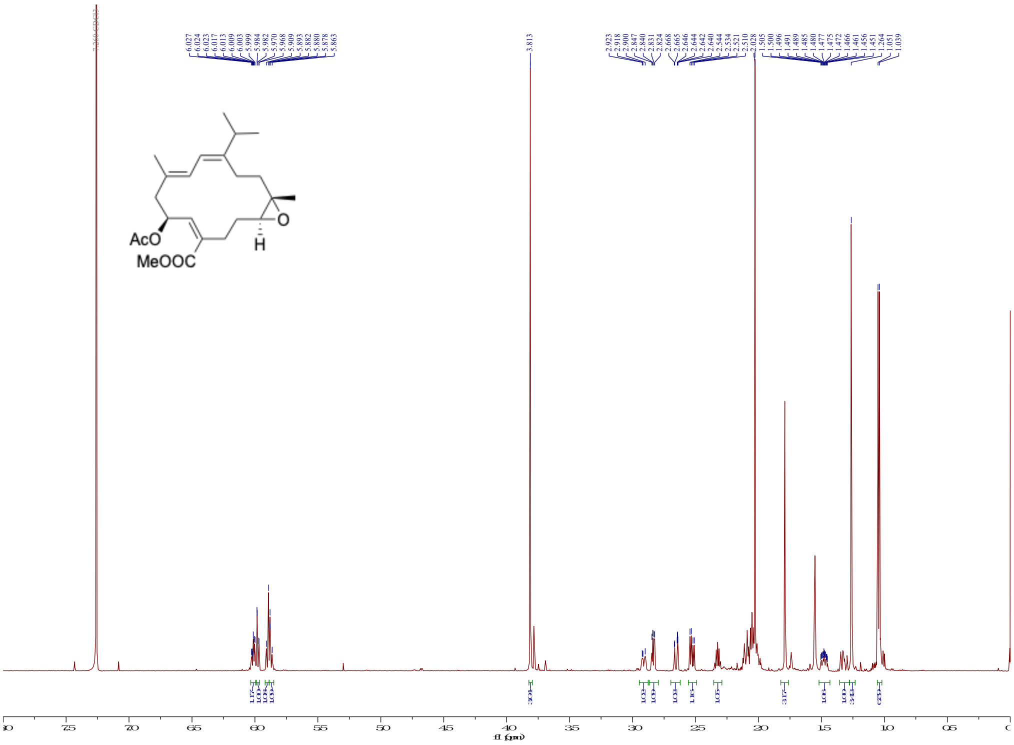

Figure S1. The ^{1}H NMR spectrum of **1**.

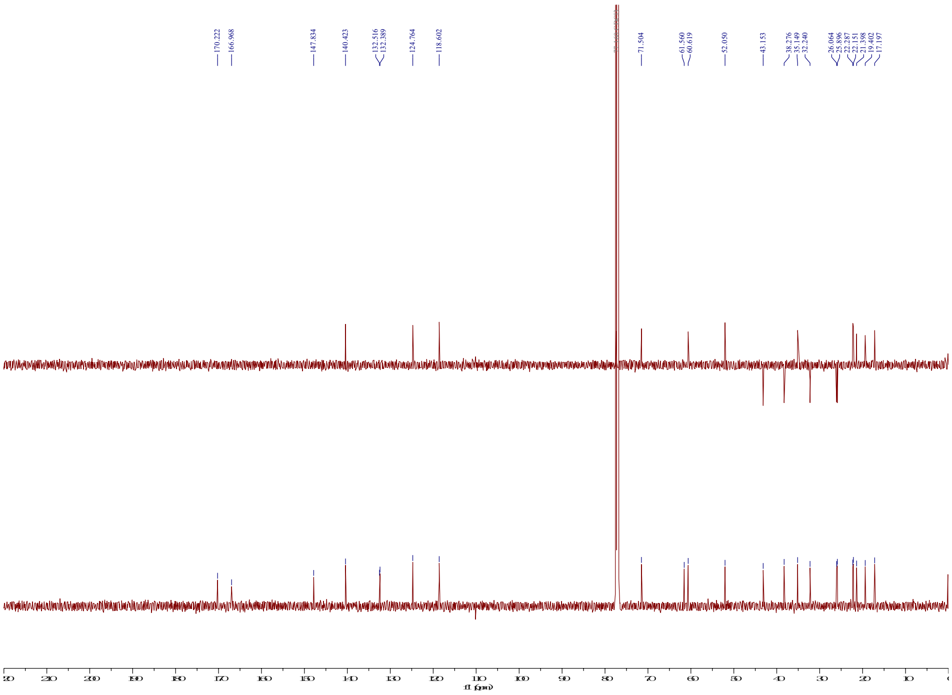

Figure S2. The ^{13}C NMR spectrum of **1**.

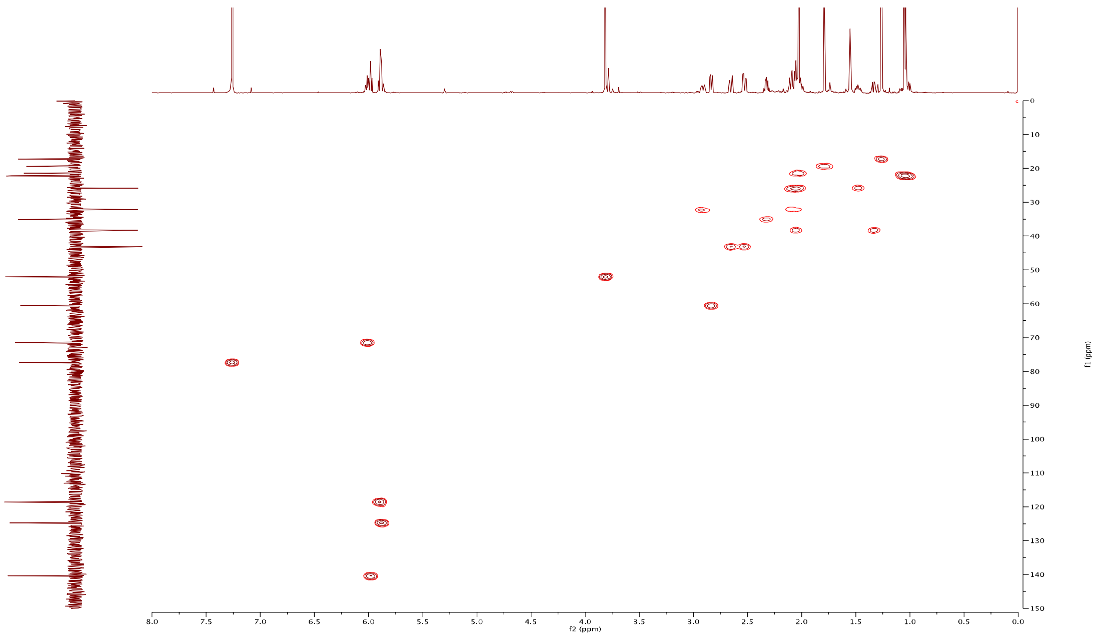

Figure S3. The HSQC spectrum of **1**.

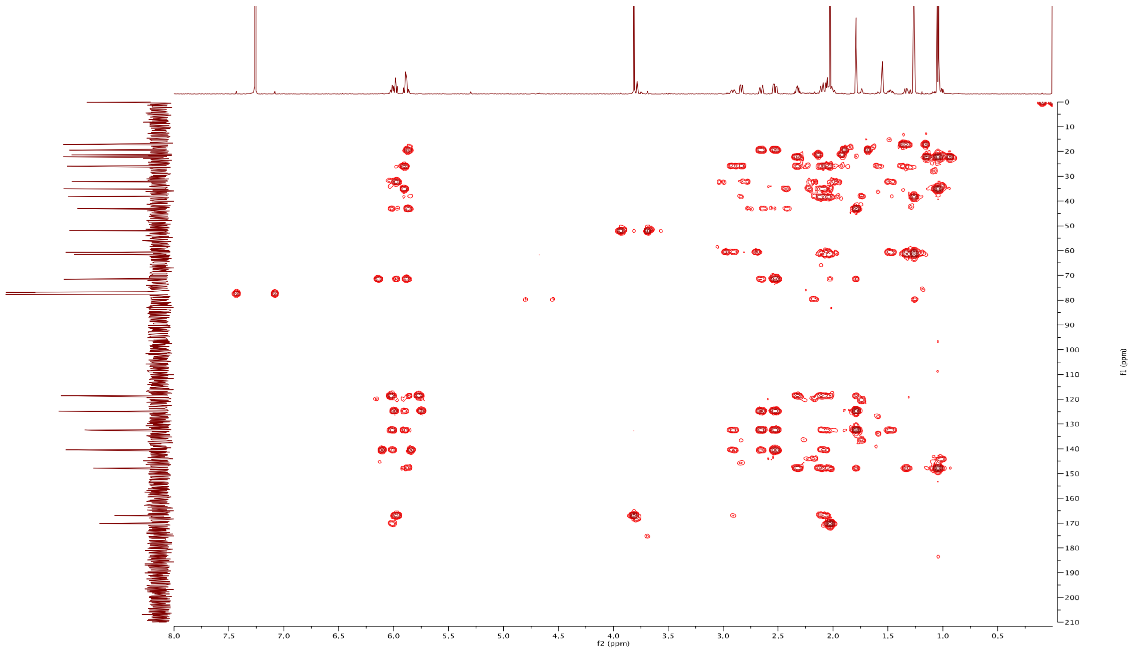

Figure S4. The HMBC spectrum of **1**.

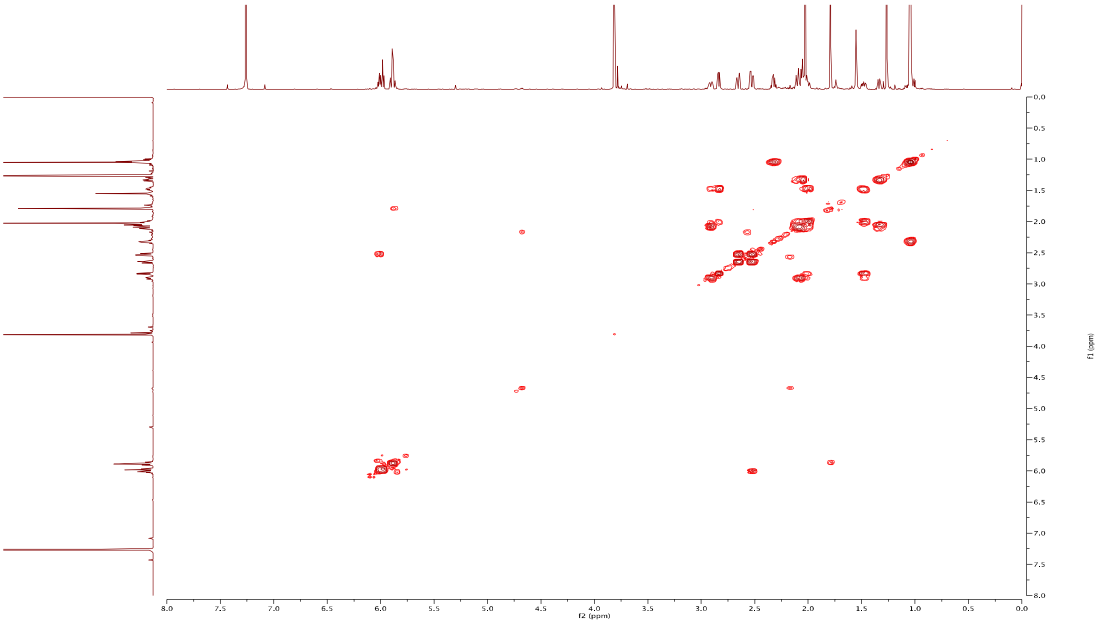

Figure S5. The ^{1}H–^{1}H COSY spectrum of **1**.

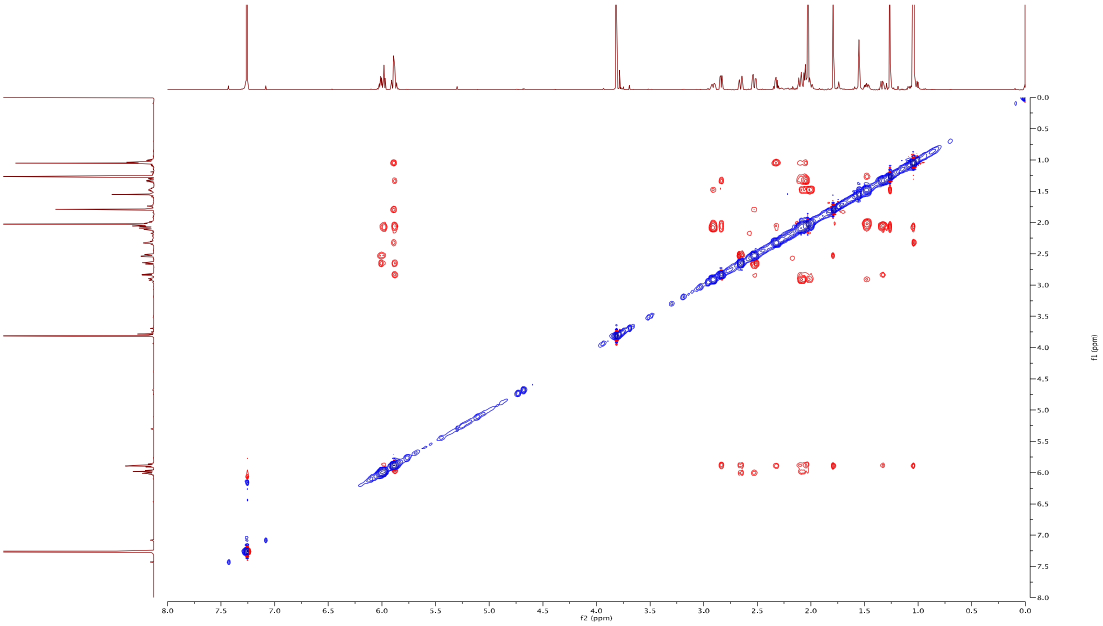

Figure S6. The NOESY spectrum of **1**.

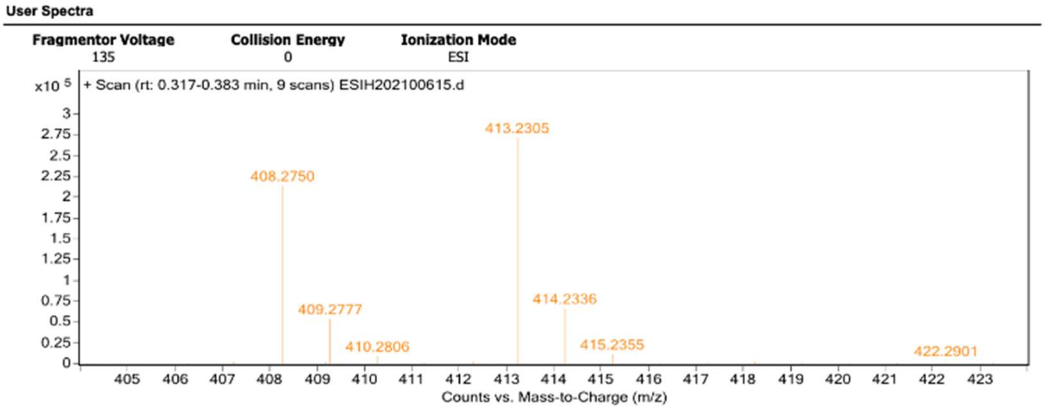

Formula Calculator Results

m/z	Calc m/z	Diff (mDa)	Diff (ppm)	Ion Formula	Ion
413.2305	413.2298	-0.69	-1.66	C23 H34 Na O5	(M+Na)+

--- End Of Report ---

Figure S7. The HR-ESIMS spectrum of **1**.

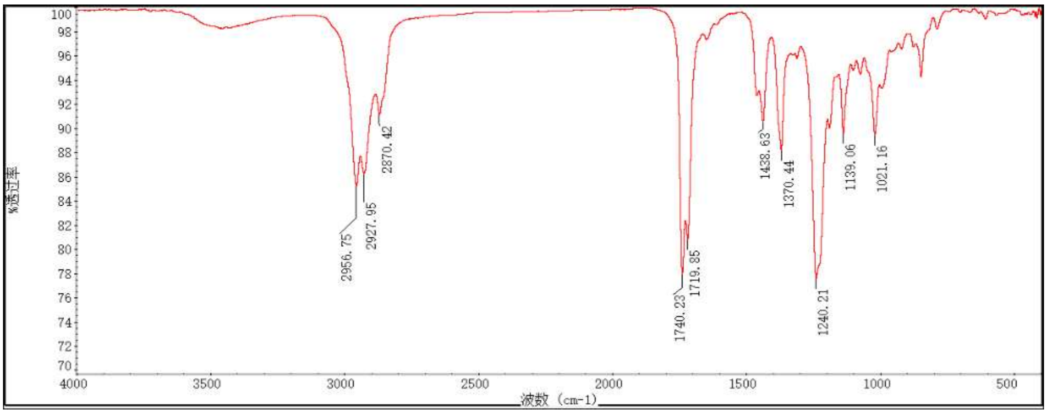

Figure S8. The IR spectrum of **1**.

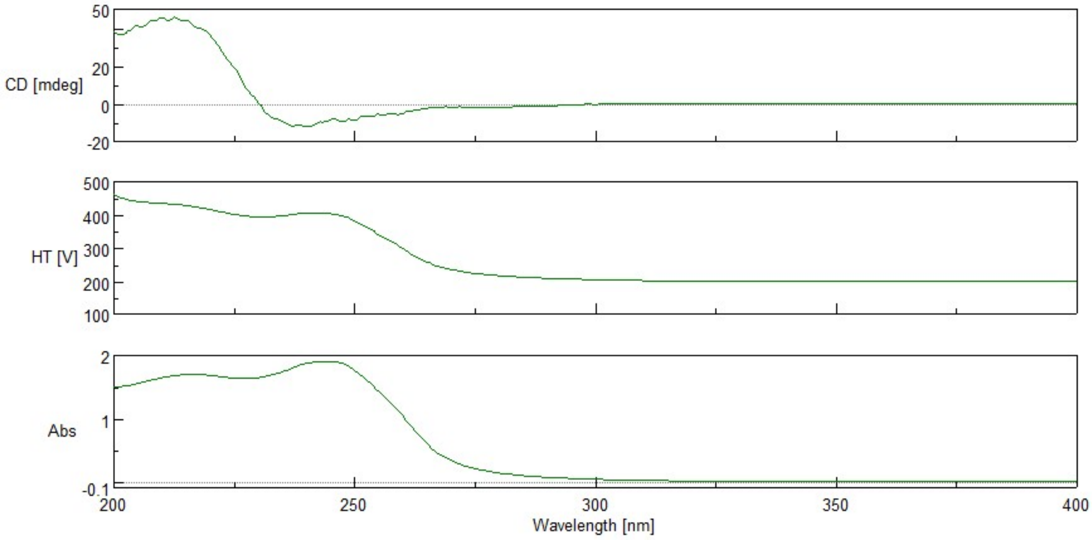

Figure S9. The ECD and UV spectra of **1**.

2. Original spectra of 2

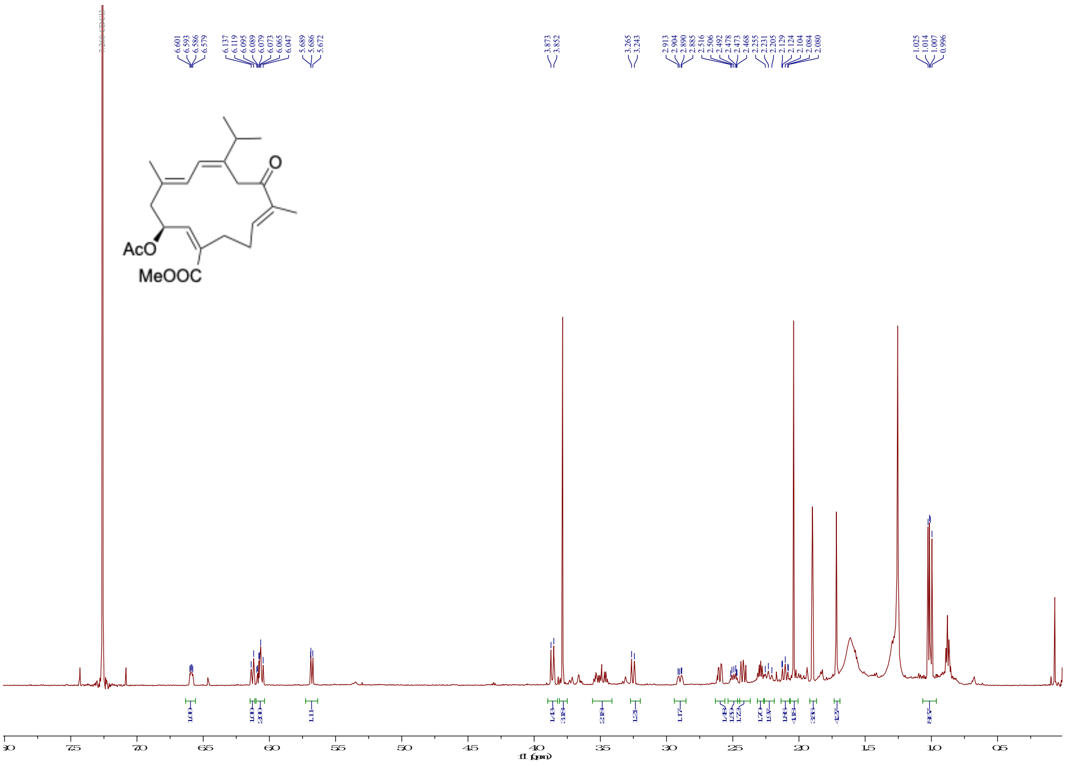

Figure S10. The ^{1}H NMR spectrum of **2**.

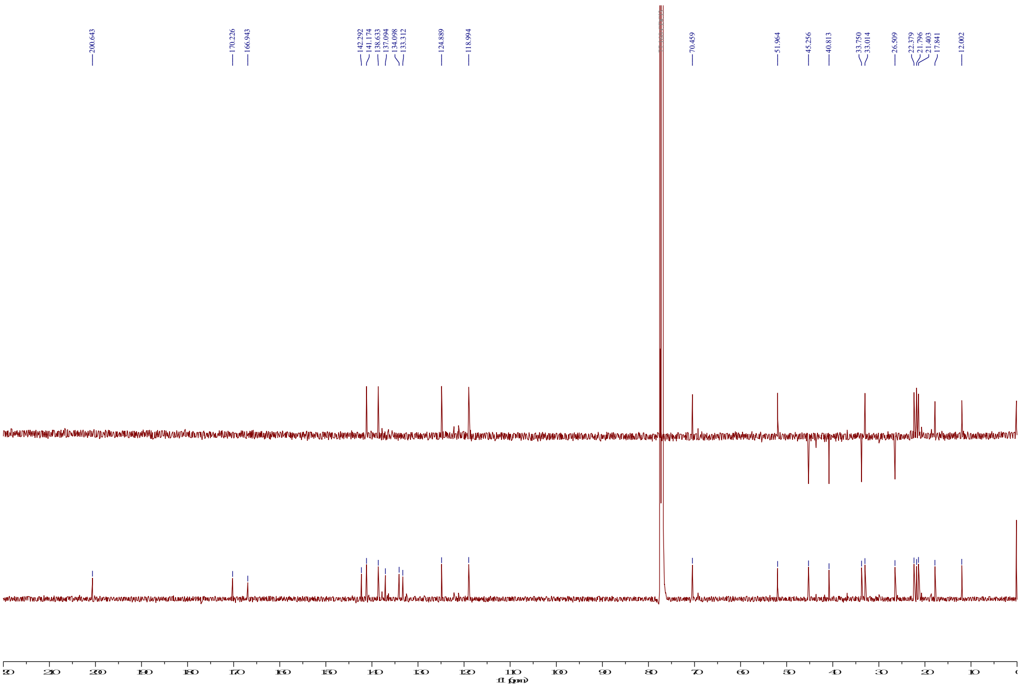

Figure S11. The ^{13}C NMR spectrum of **2**.

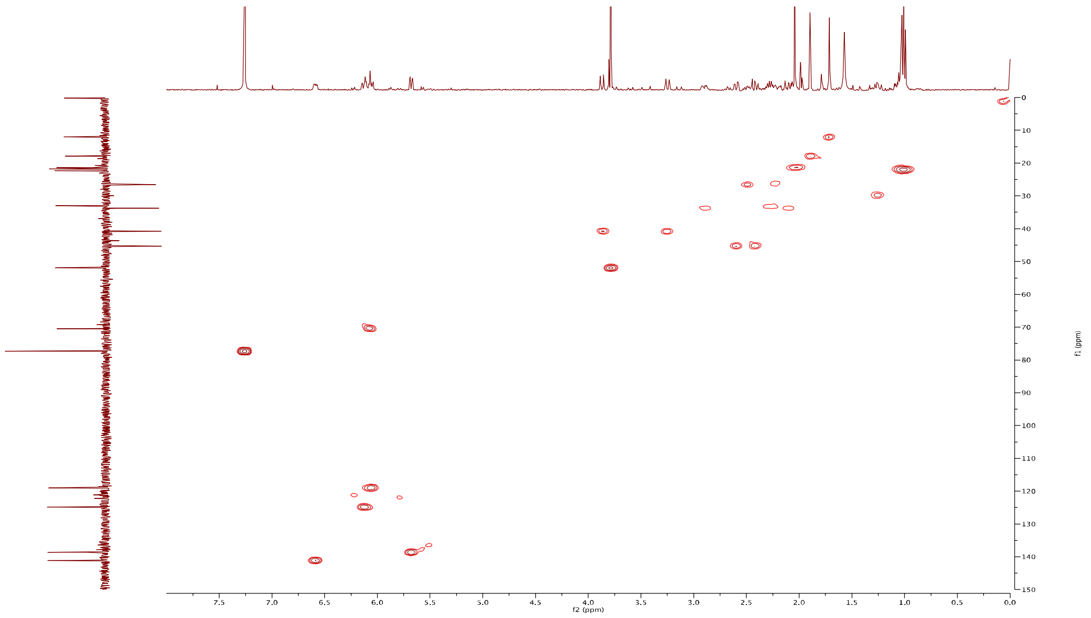

Figure S12. The HSQC spectrum of **2**.

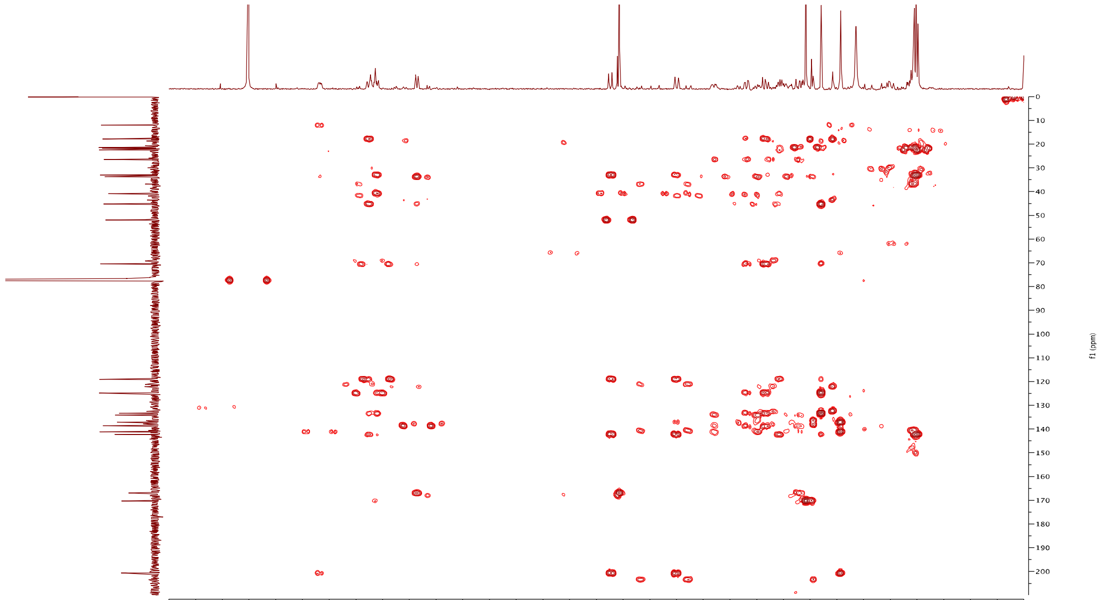

Figure S13. The HMBC spectrum of **2**.

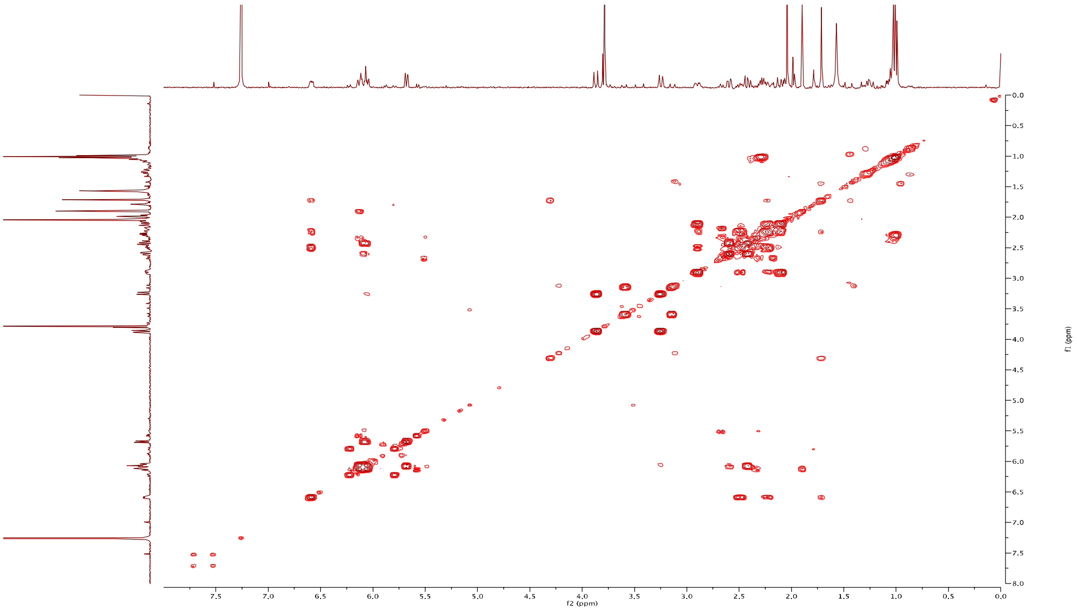

Figure S14. The $^1H–^1H$ COSY spectrum of **2**.

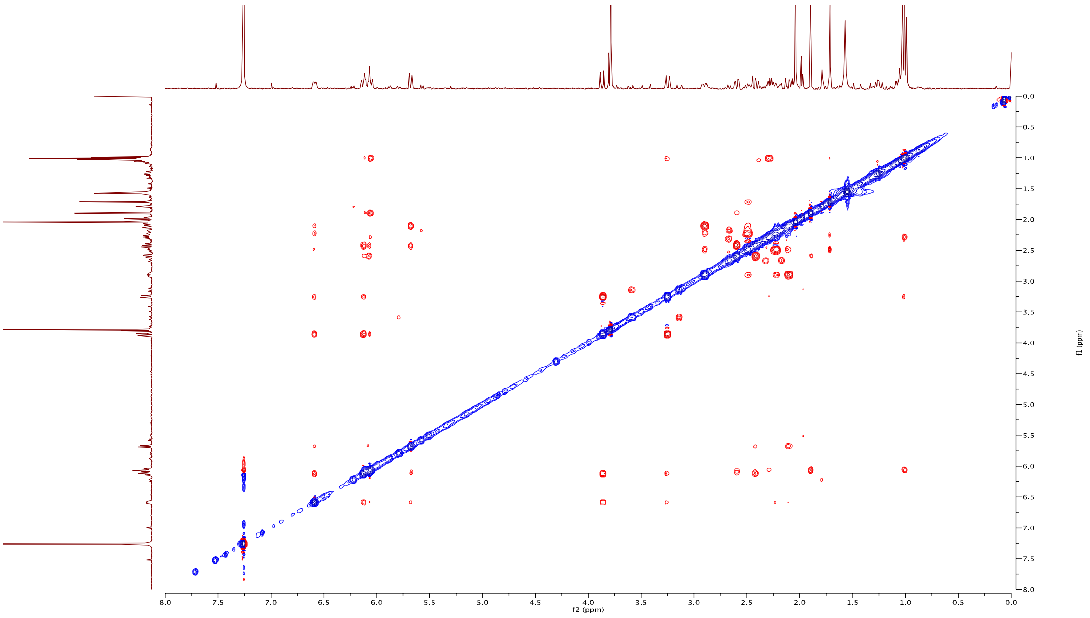

Figure S15. The NOESY spectrum of **2**.

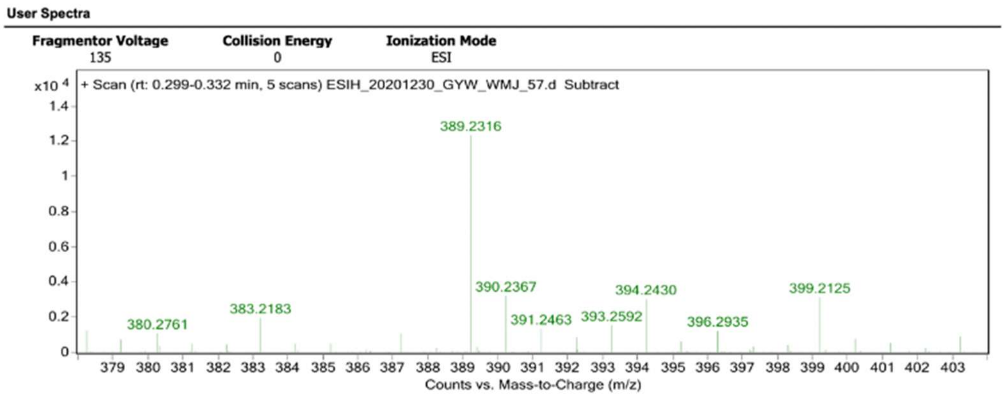

Formula Calculator Results

m/z	Calc m/z	Diff (mDa)	Diff (ppm)	Ion Formula	Ion
389.2316	389.2323	0.68	1.74	C23 H33 O5	(M+H)+

--- End Of Report ---

Figure S16. The HR-ESIMS spectrum of **2**.

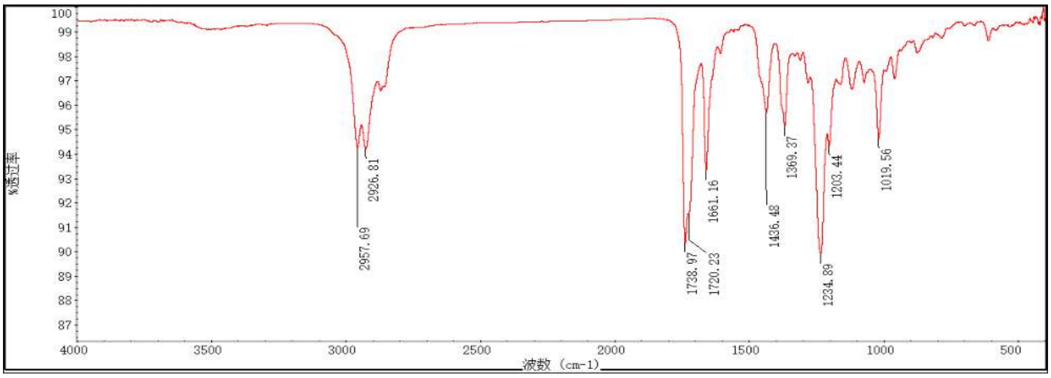

Figure S17. The IR spectrum of **2**.

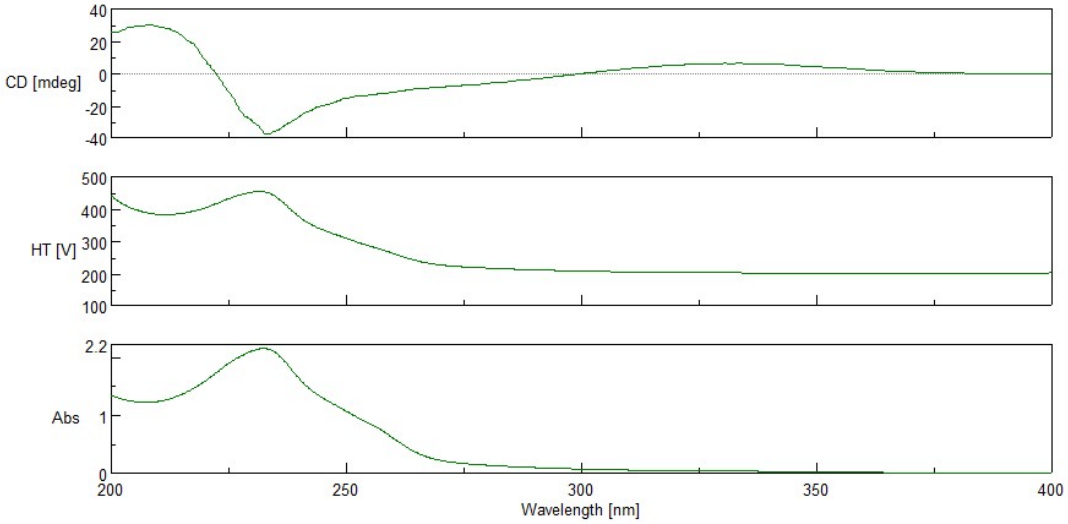

Figure S18. The ECD and UV spectra of **2**.

3. Original spectra of 3

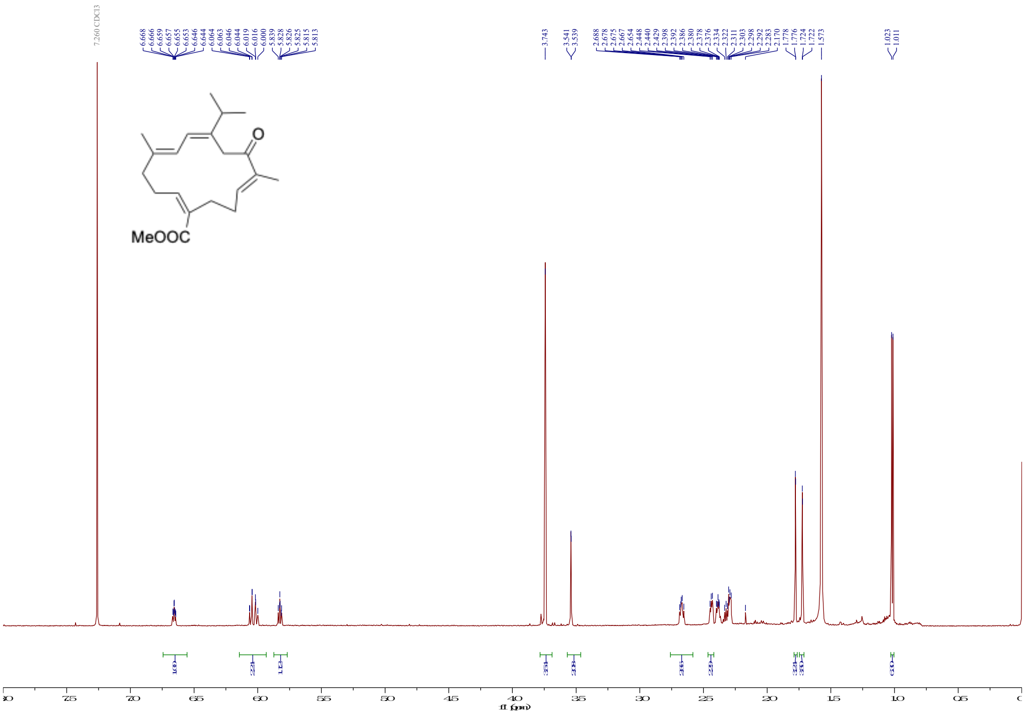

Figure S19. The ^{1}H NMR spectrum of **3**.

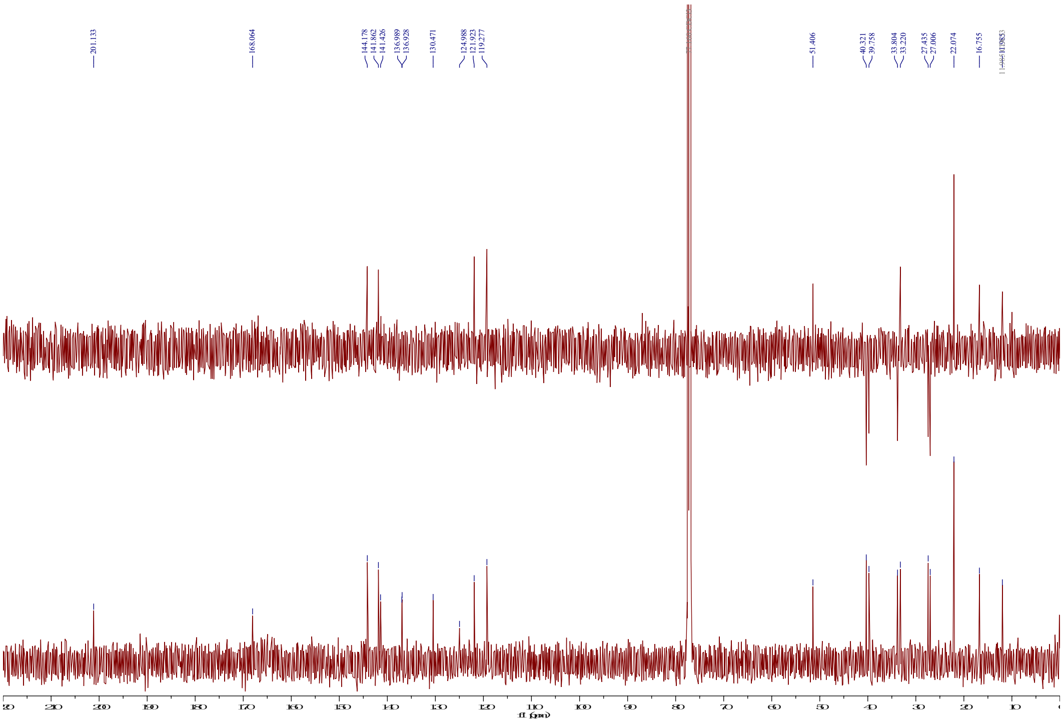

Figure S20. The ^{13}C NMR spectrum of **3**.

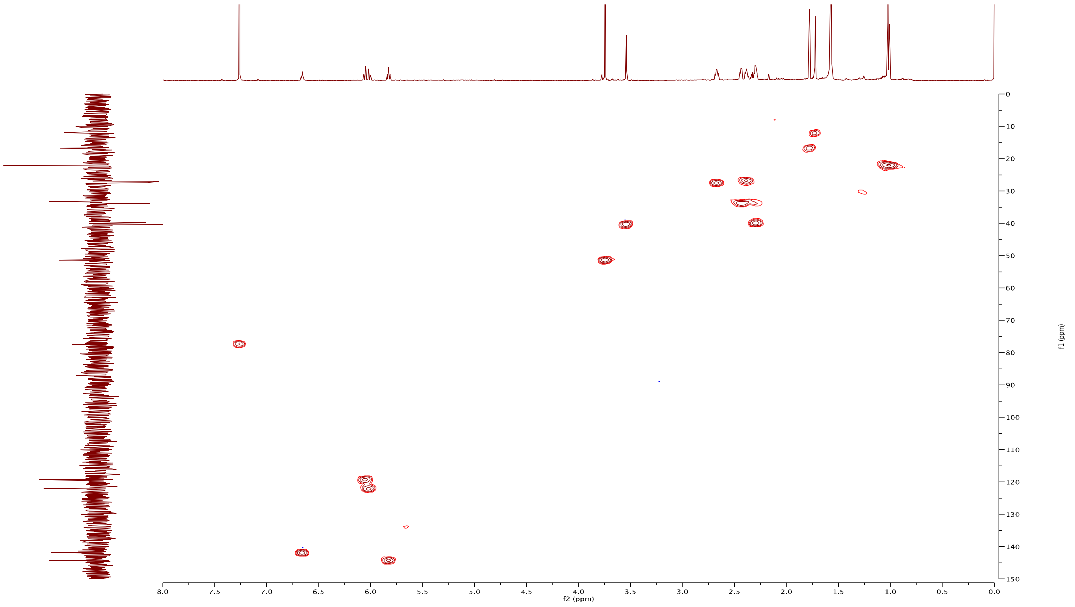

Figure S21. The HSQC spectrum of **3**.

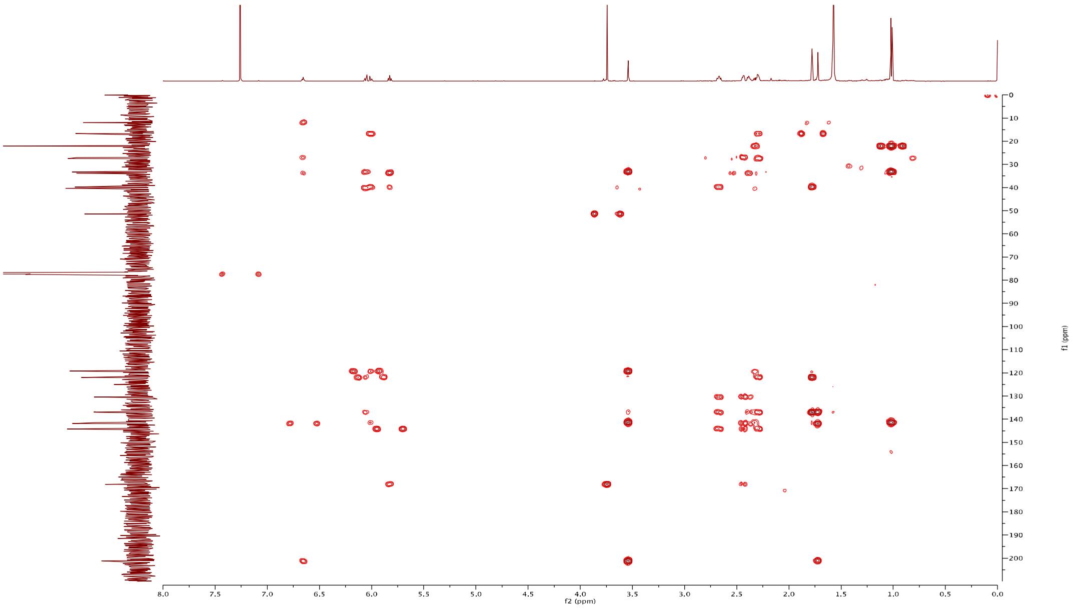

Figure S22. The HMBC spectrum of **3**.

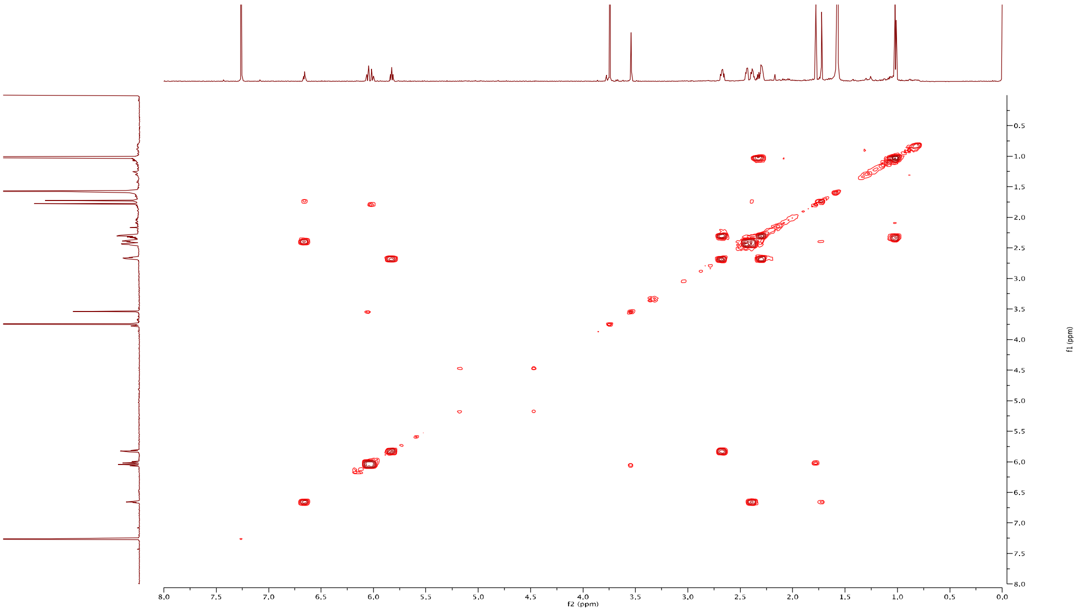

Figure S23. The ^{1}H–^{1}H COSY spectrum of **3**.

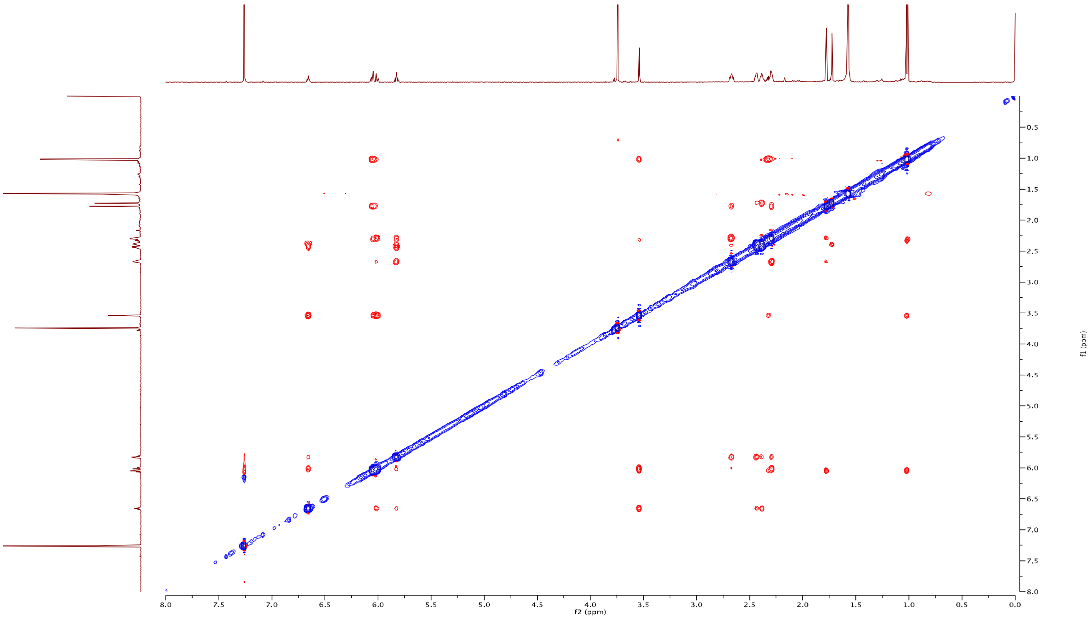

Figure S24. The NOESY spectrum of **3**.

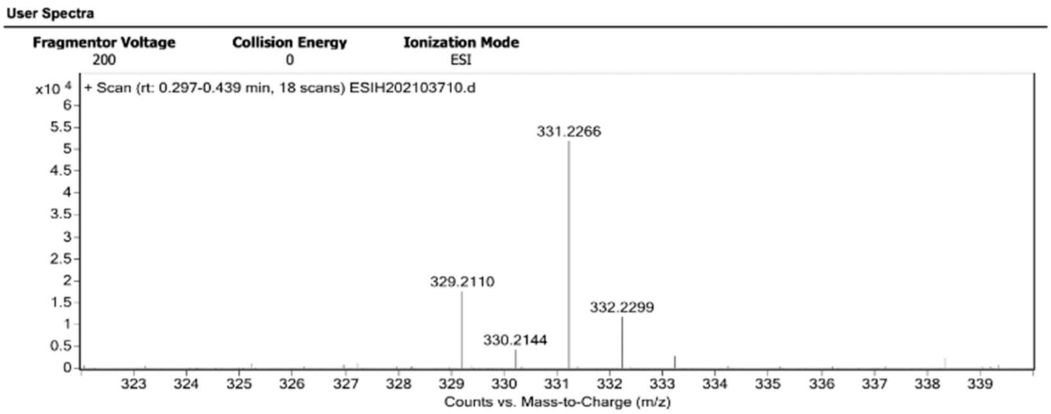

Formula Calculator Results

m/z	Calc m/z	Diff (mDa)	Diff (ppm)	Ion Formula	Ion
331.2266	331.2268	0.18	0.54	C21 H31 O3	(M+H)+

--- End Of Report ---

Figure S25. The HR-ESIMS spectrum of **3**.

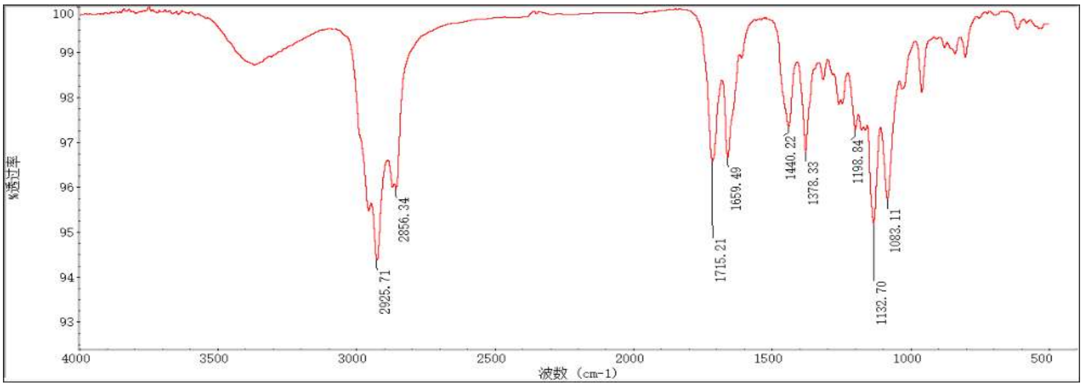

Figure S26. The IR spectrum of **3**.

4. Original spectra of 4

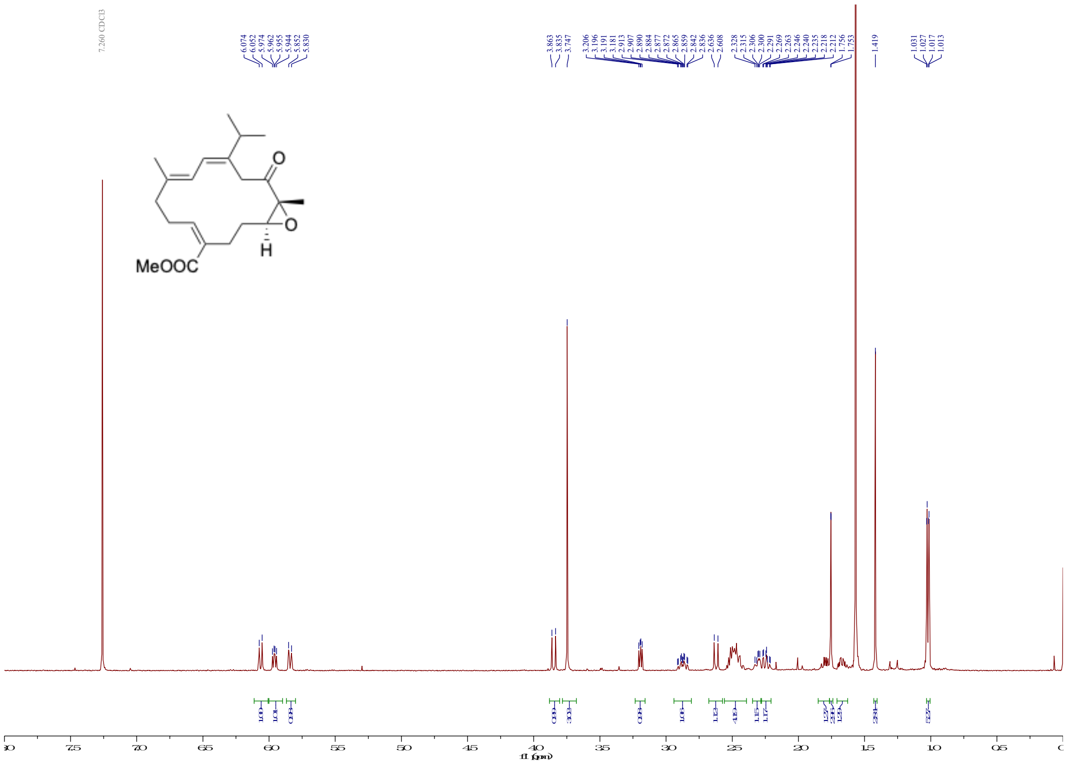

Figure S27. The ^{1}H NMR spectrum of **4**.

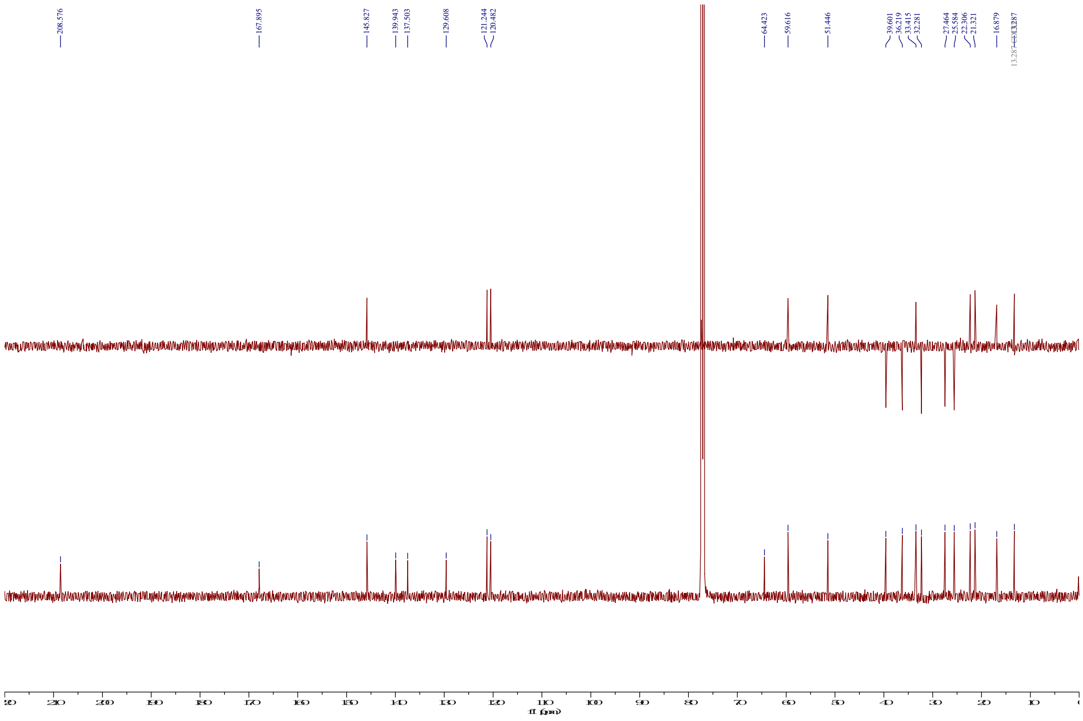

Figure S28. The ^{13}C NMR spectrum of **4**.

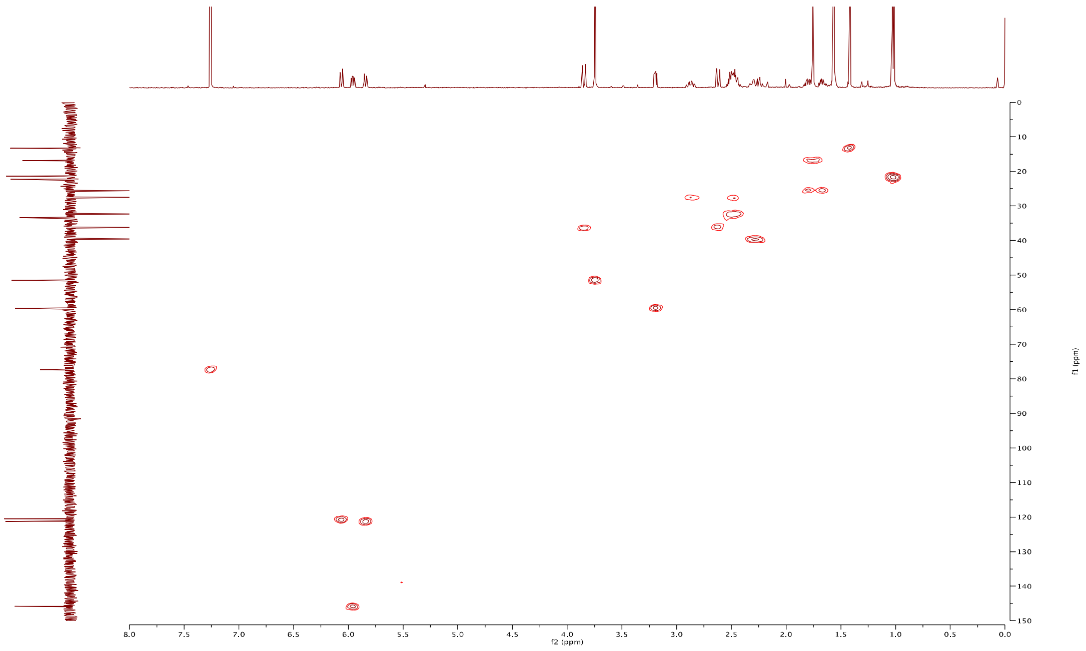

Figure S29. The HSQC spectrum of **4**.

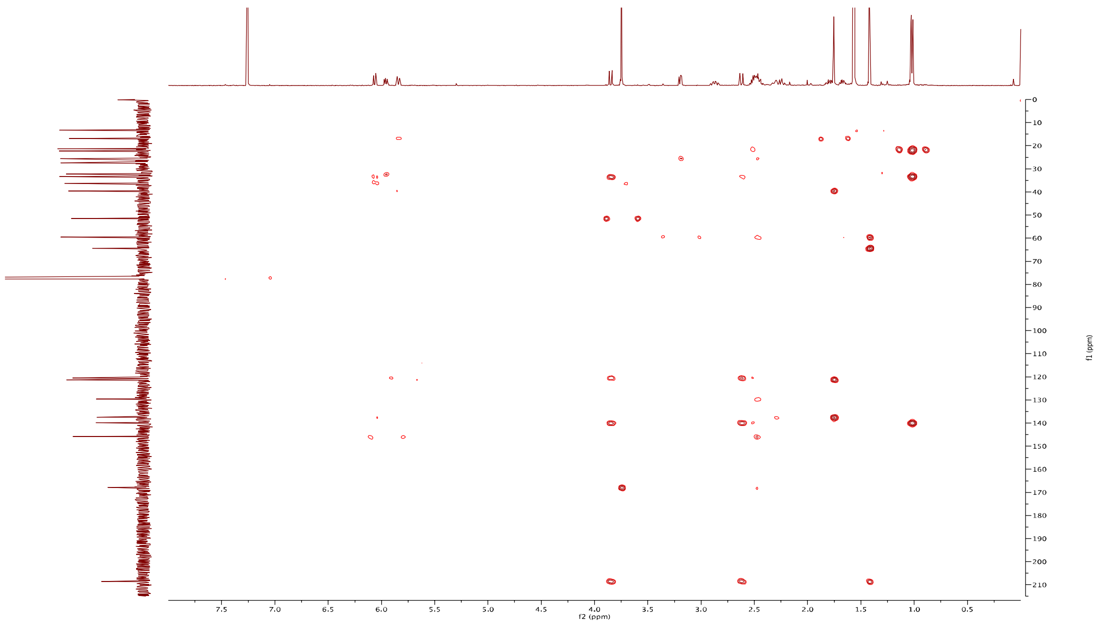

Figure S30. The HMBC spectrum of **4**.

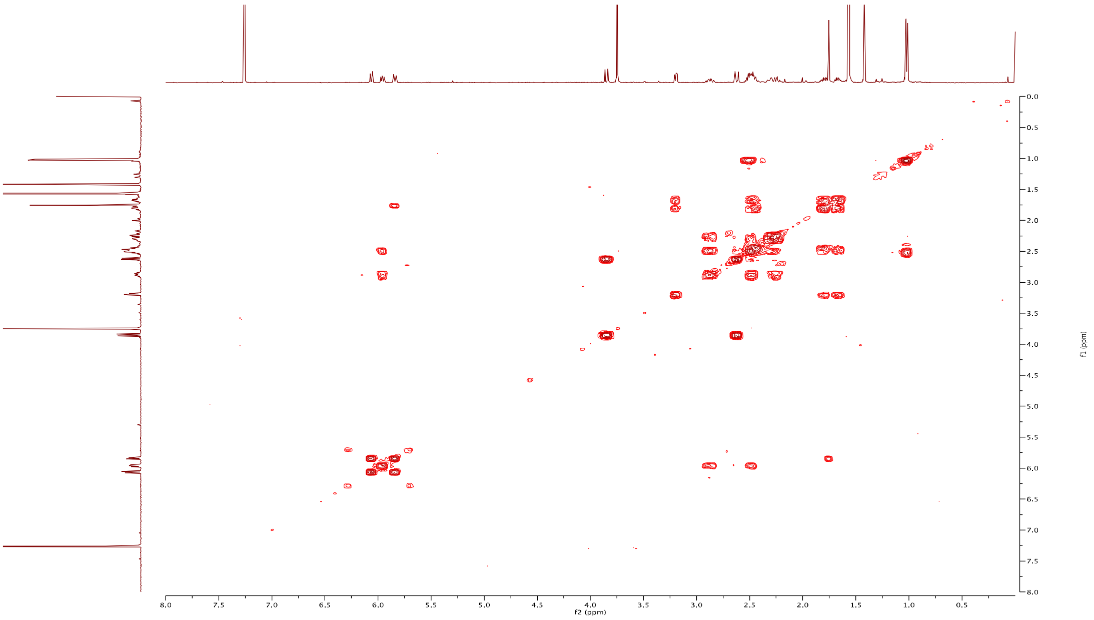

Figure S31. The ^{1}H–^{1}H COSY spectrum of **4**.

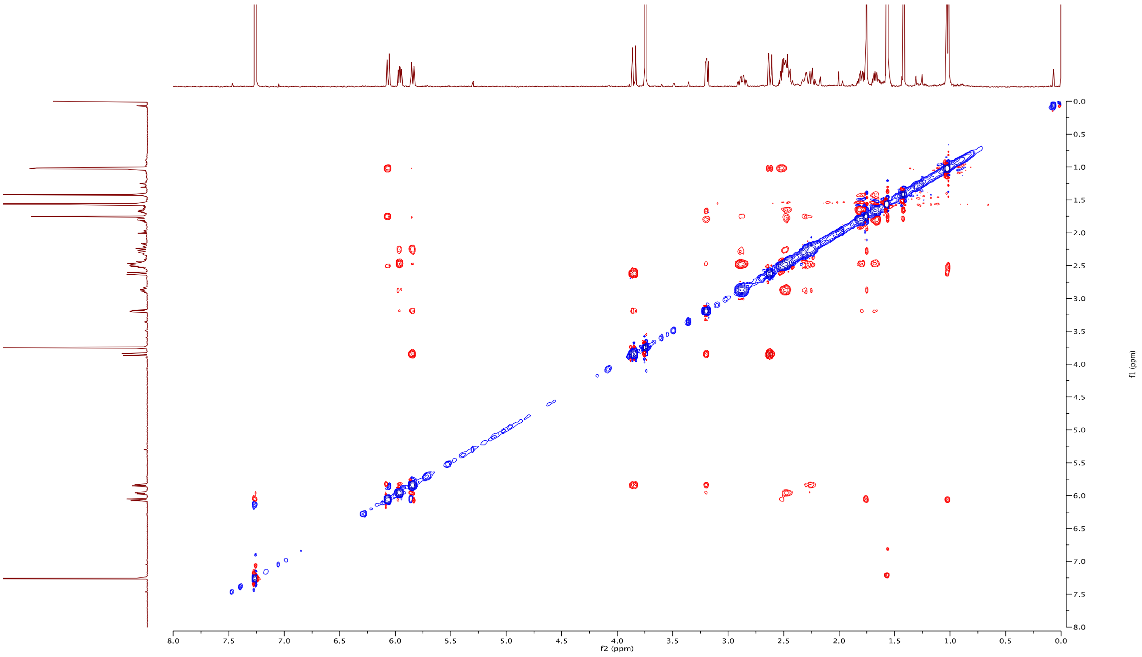

Figure S32. The NOESY spectrum of **4**.

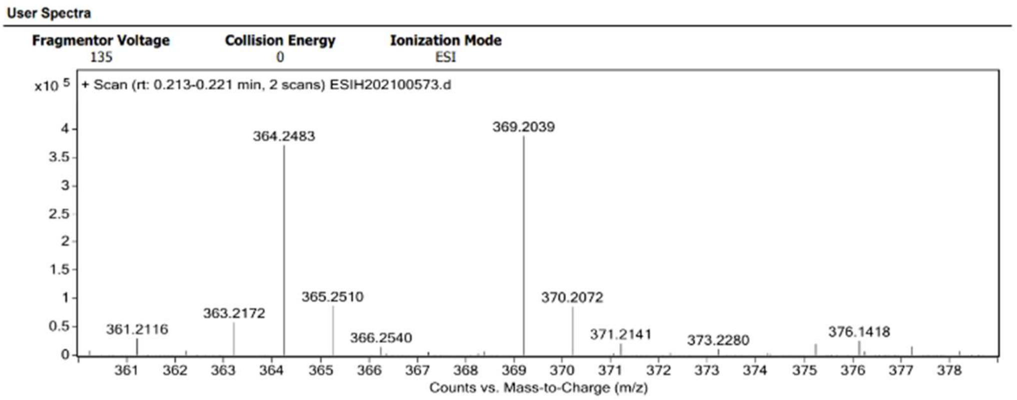

Formula Calculator Results

m/z	Calc m/z	Diff (mDa)	Diff (ppm)	Ion Formula	Ion
369.2039	369.2036	-0.29	-0.78	C21 H30 Na O4	(M+Na)+

--- End Of Report ---

Figure S33. The HR-ESIMS spectrum of **4**.

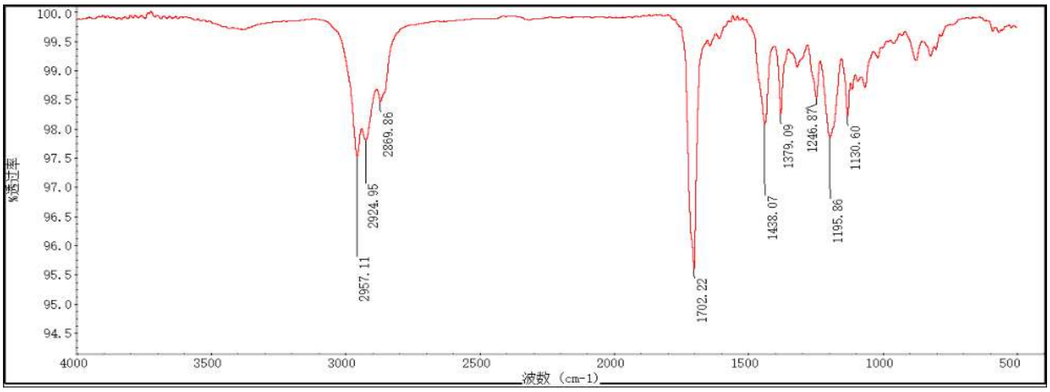

Figure S34. The IR spectrum of **4**.

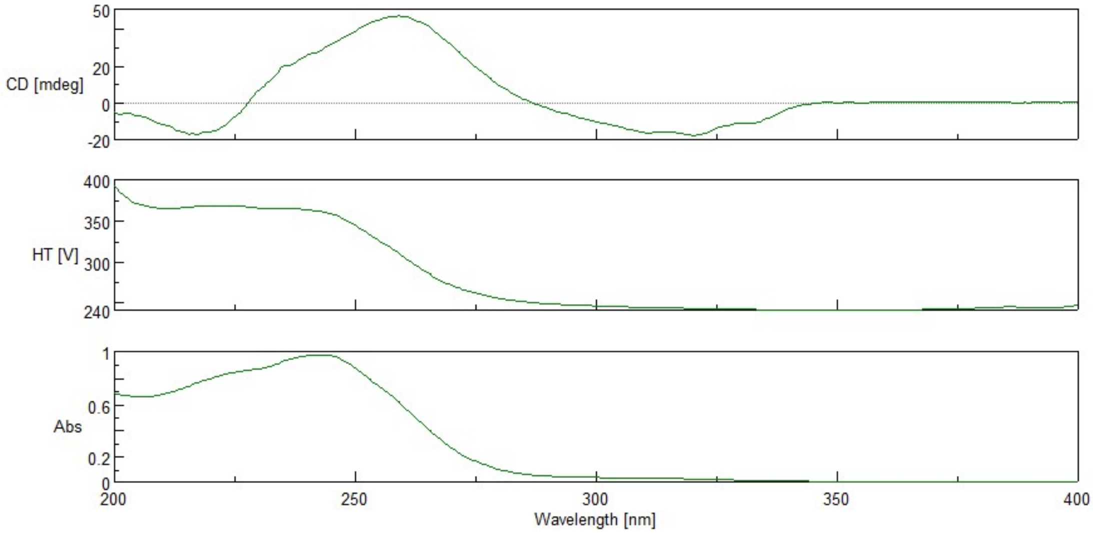

Figure S35. The ECD and UV spectra of **4**.

5. Original spectra of 5

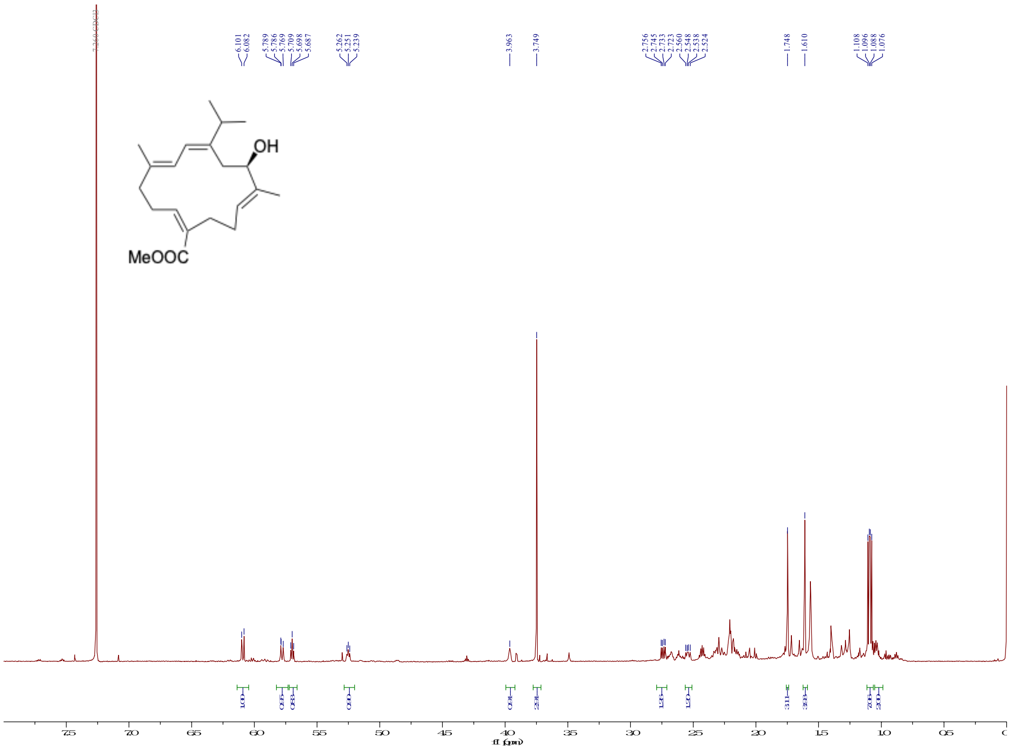

Figure S36. The ^{1}H NMR spectrum of **5**.

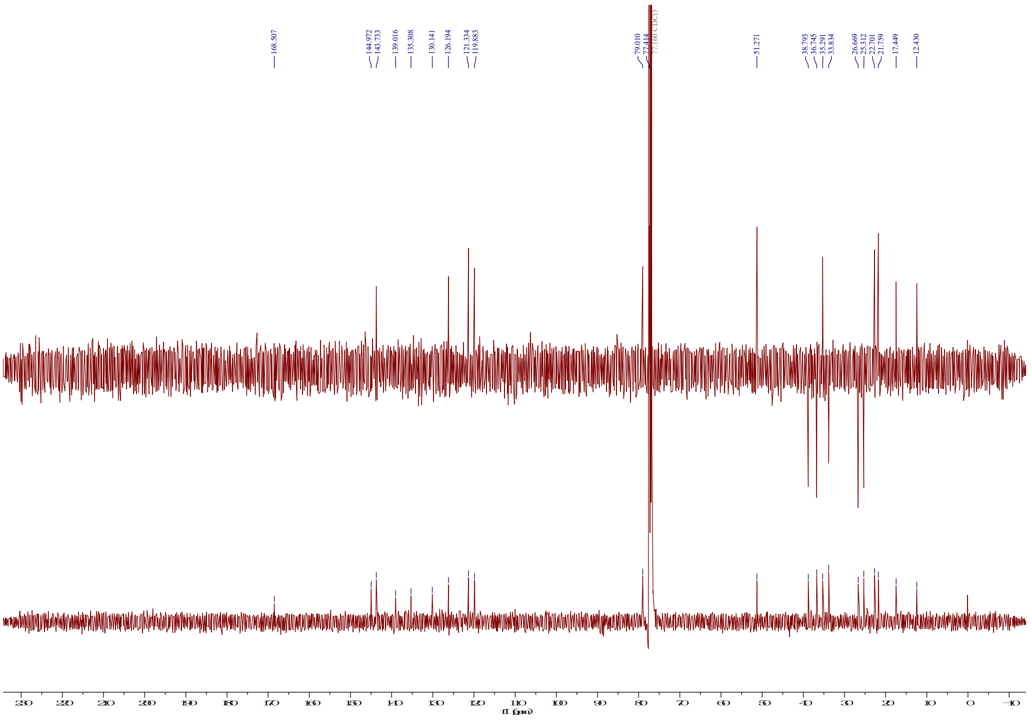

Figure S37. The ^{13}C NMR spectrum of **5**.

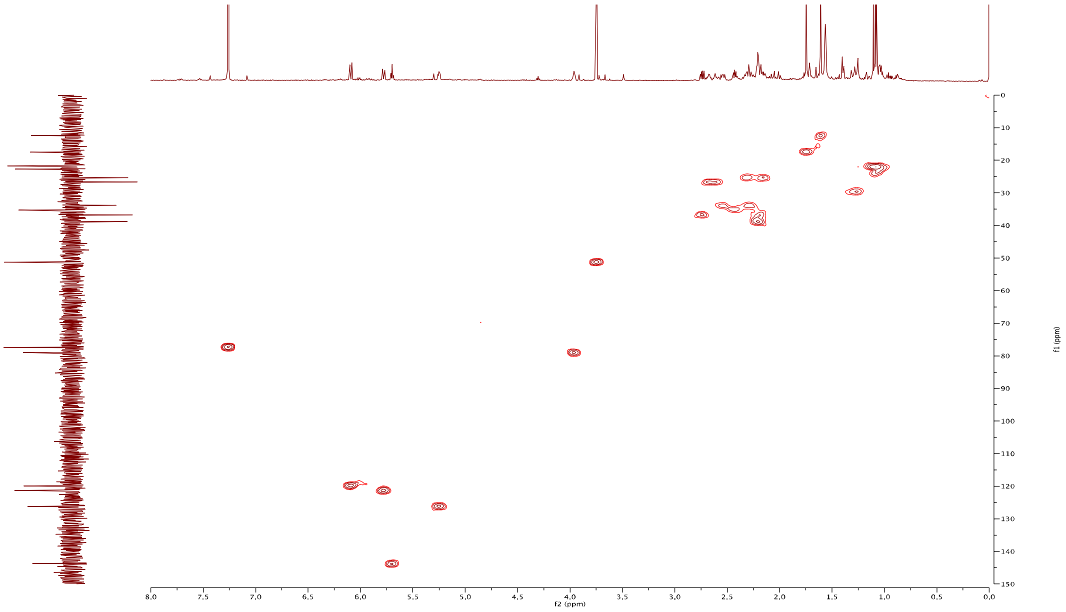

Figure S38. The HSQC spectrum of **5**.

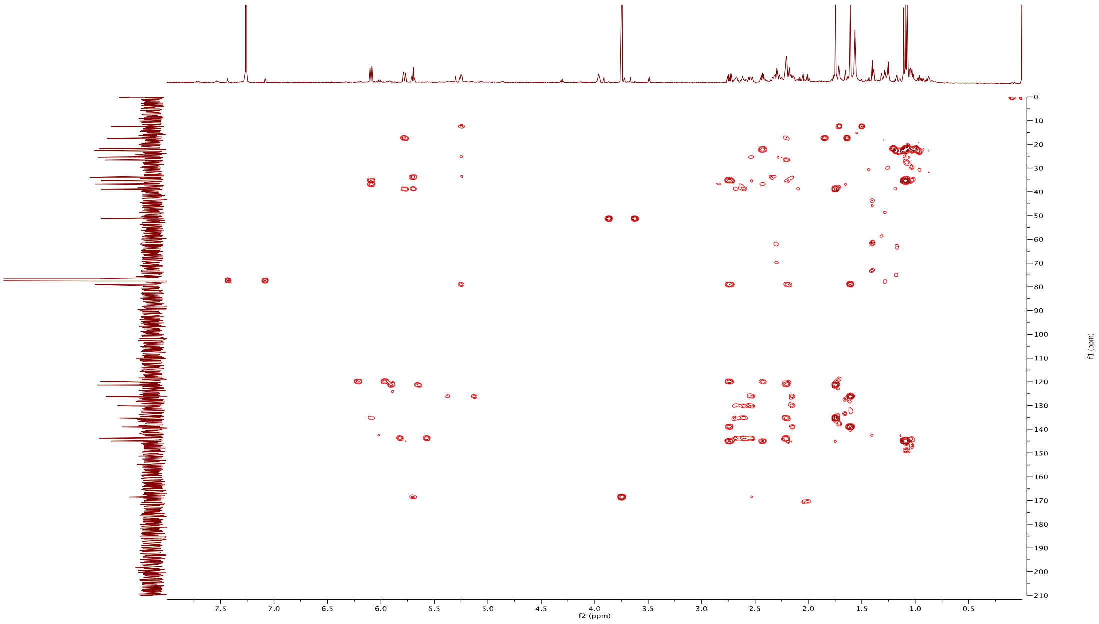

Figure S39. The HMBC spectrum of **5**.

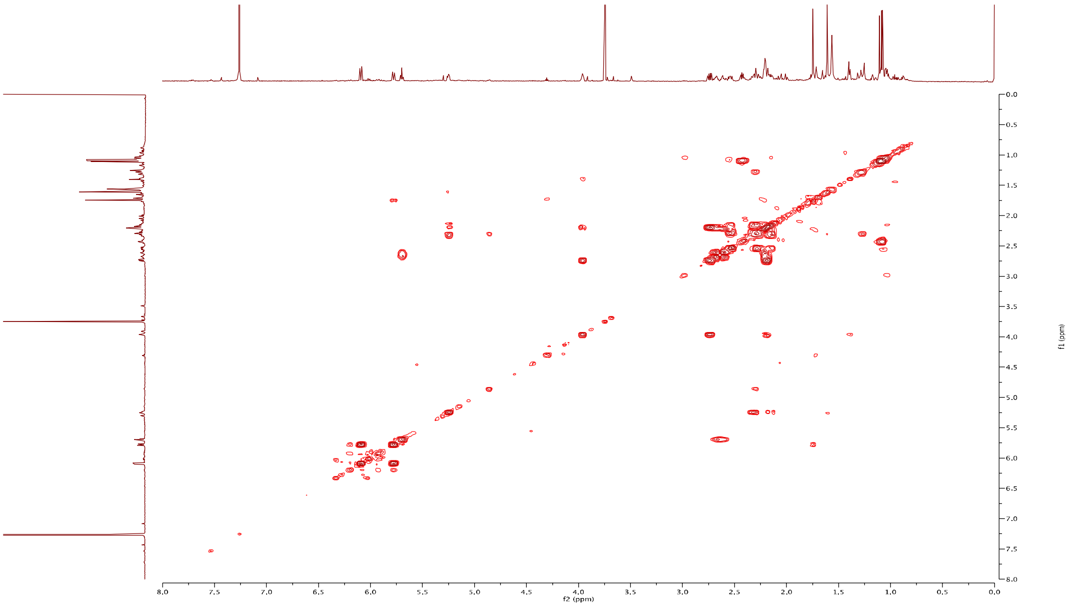

Figure S40. The ^{1}H–^{1}H COSY spectrum of **5**.

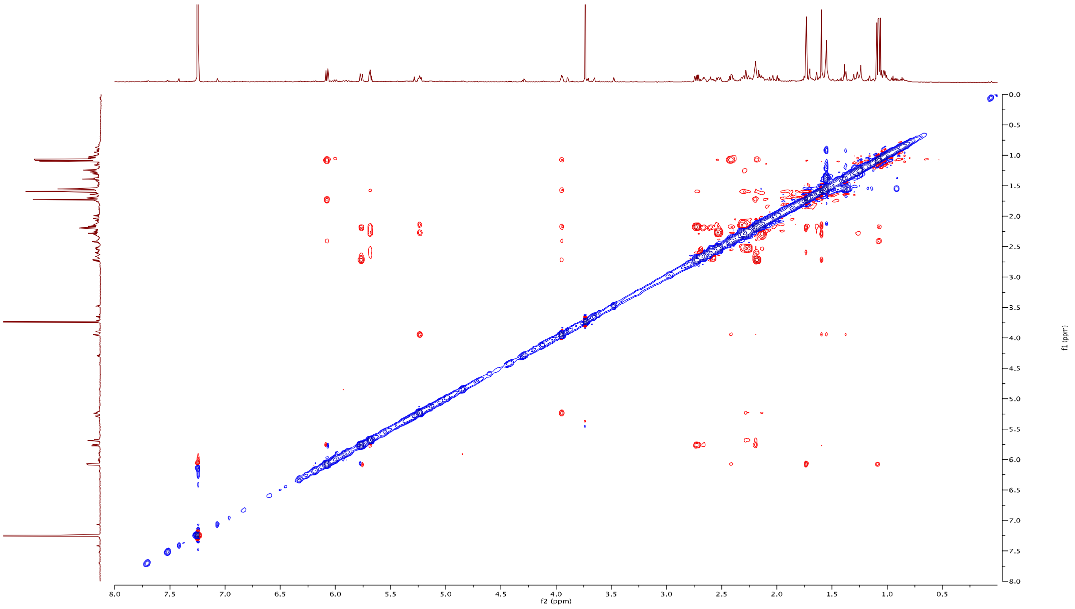

Figure S41. The NOESY spectrum of **5**.

User Spectra

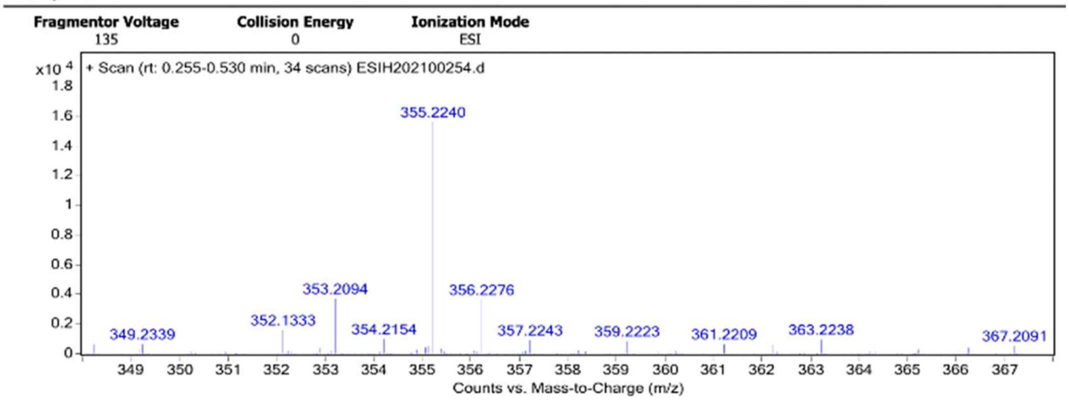

Formula Calculator Results

m/z	Calc m/z	Diff (mDa)	Diff (ppm)	Ion Formula	Ion
355.224	355.2244	0.34	0.96	C21 H32 Na O3	(M+Na)+

--- End Of Report ---

Figure S42. The HR-ESIMS spectrum of **5**.

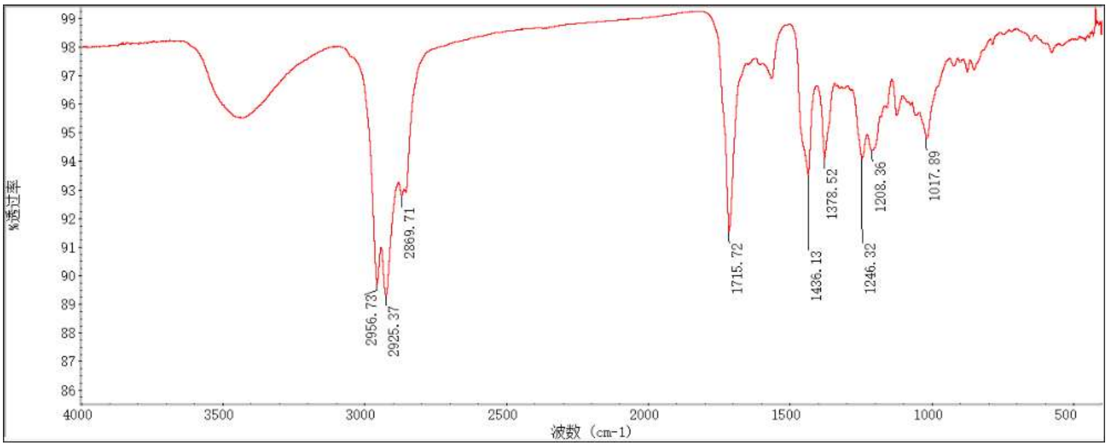

Figure S43. The IR spectrum of **5**.

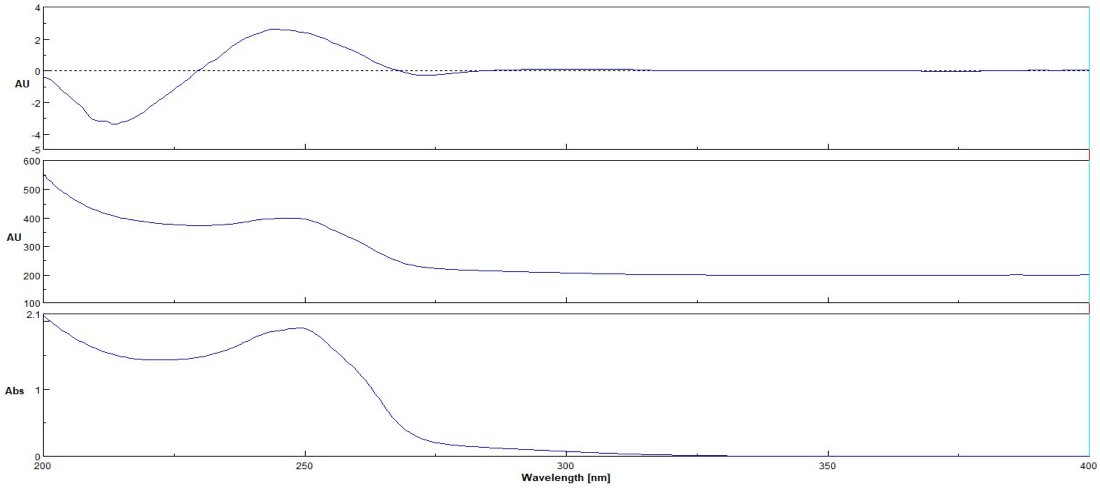

Figure S44. The ECD and UV spectra of **5**.

6. Original spectra of 6

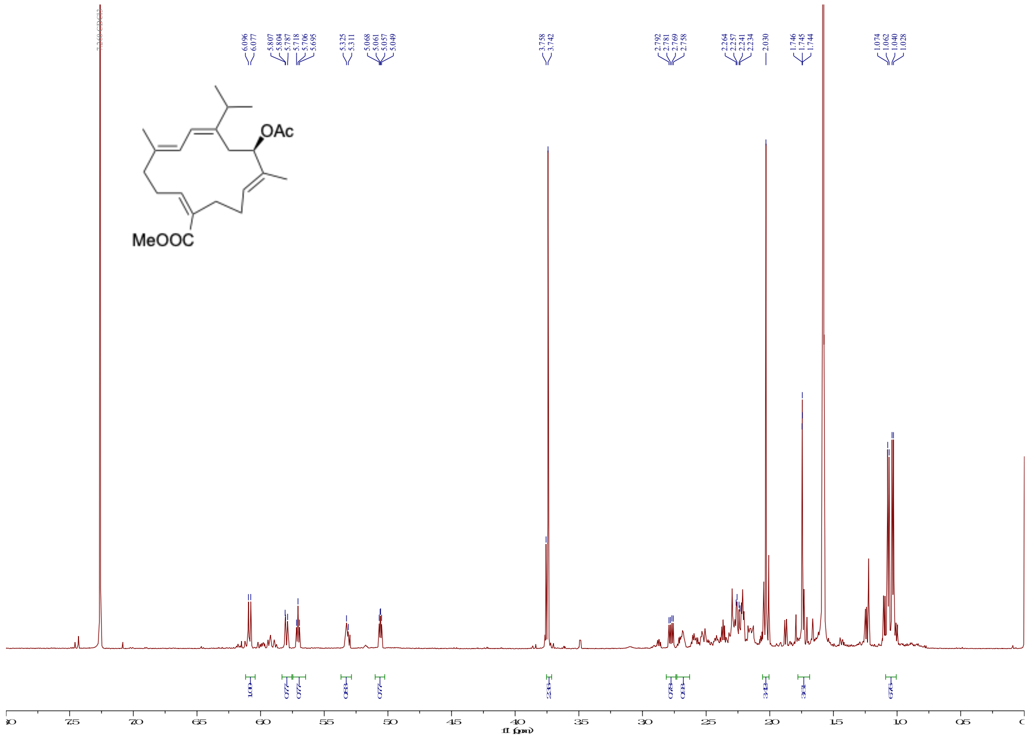

Figure S45. The ^1H NMR spectrum of **6**.

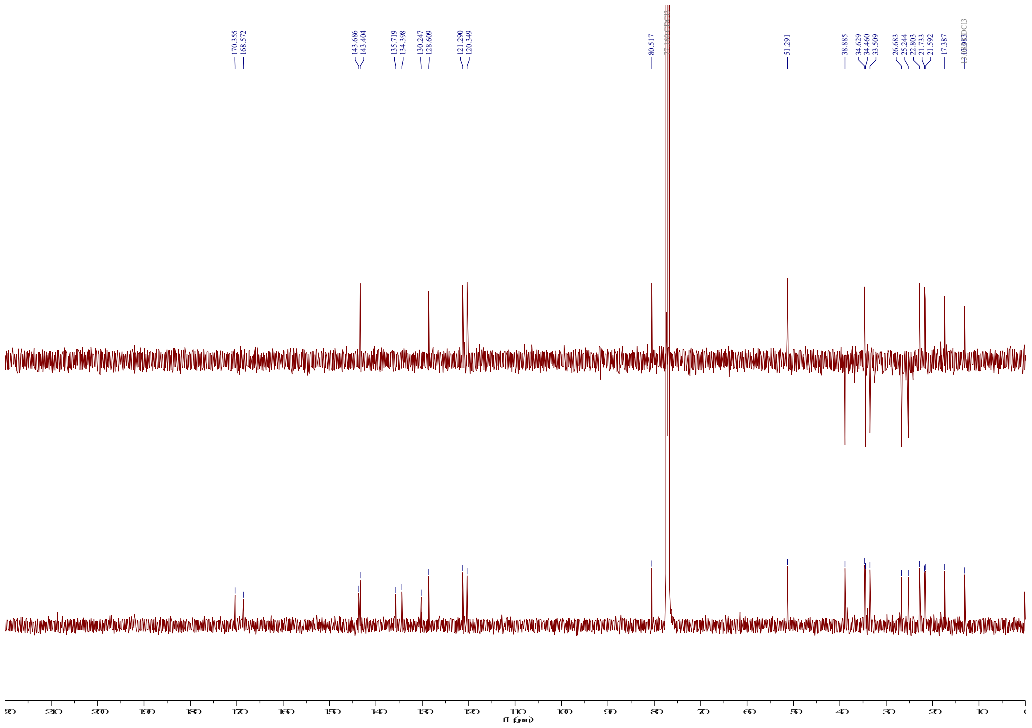

Figure S46. The ^{13}C NMR spectrum of **6**.

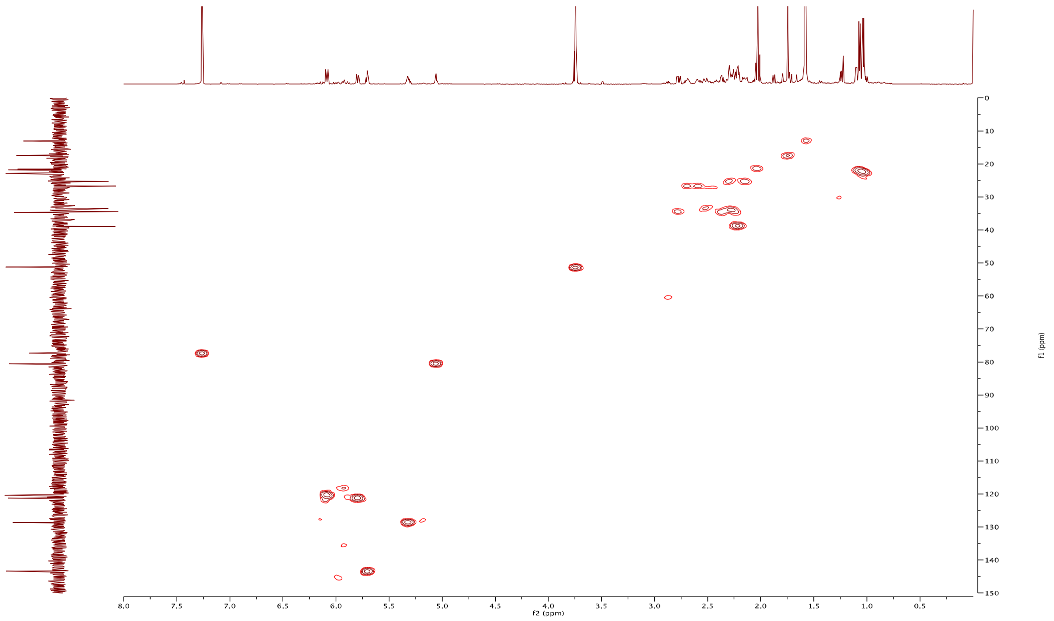

Figure S47. The HSQC spectrum of **6**.

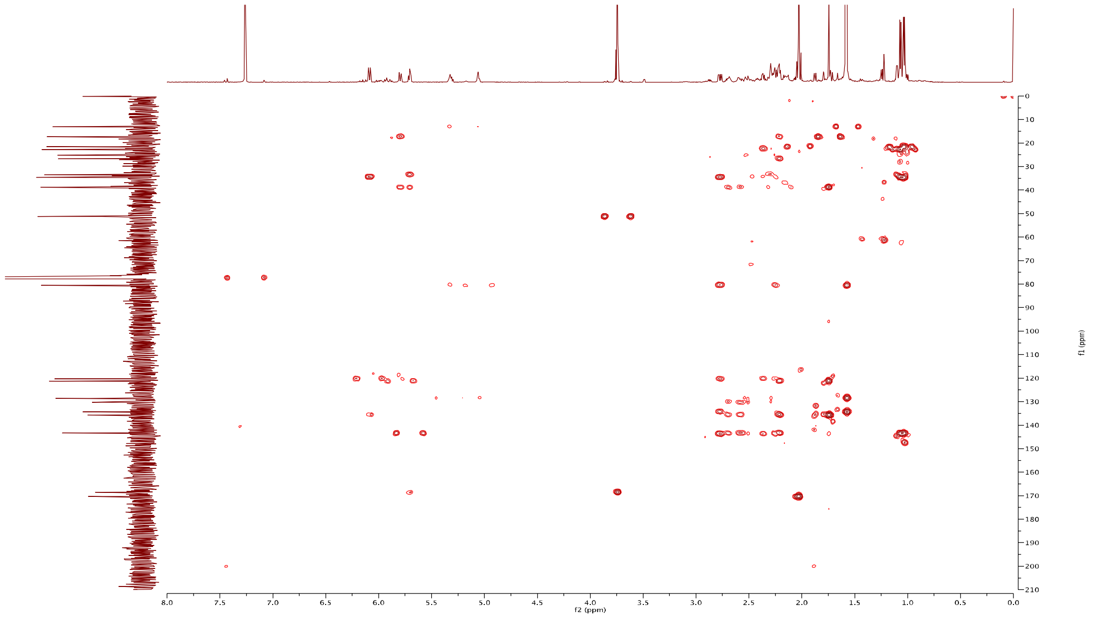

Figure S48. The HMBC spectrum of **6**.

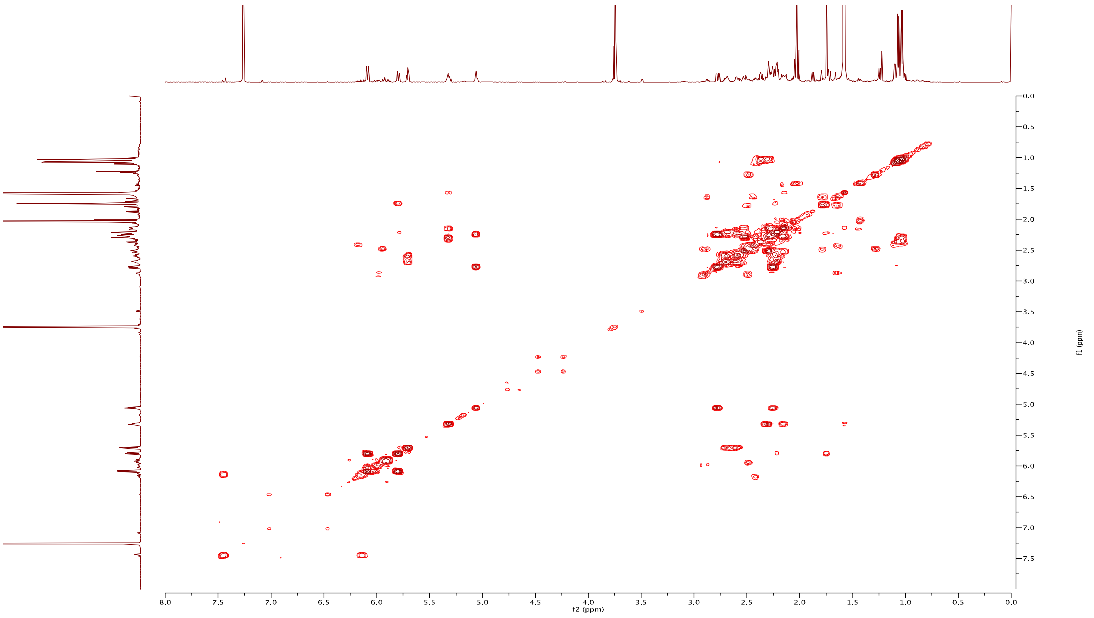

Figure S49. The ^{1}H–^{1}H COSY spectrum of **6**.

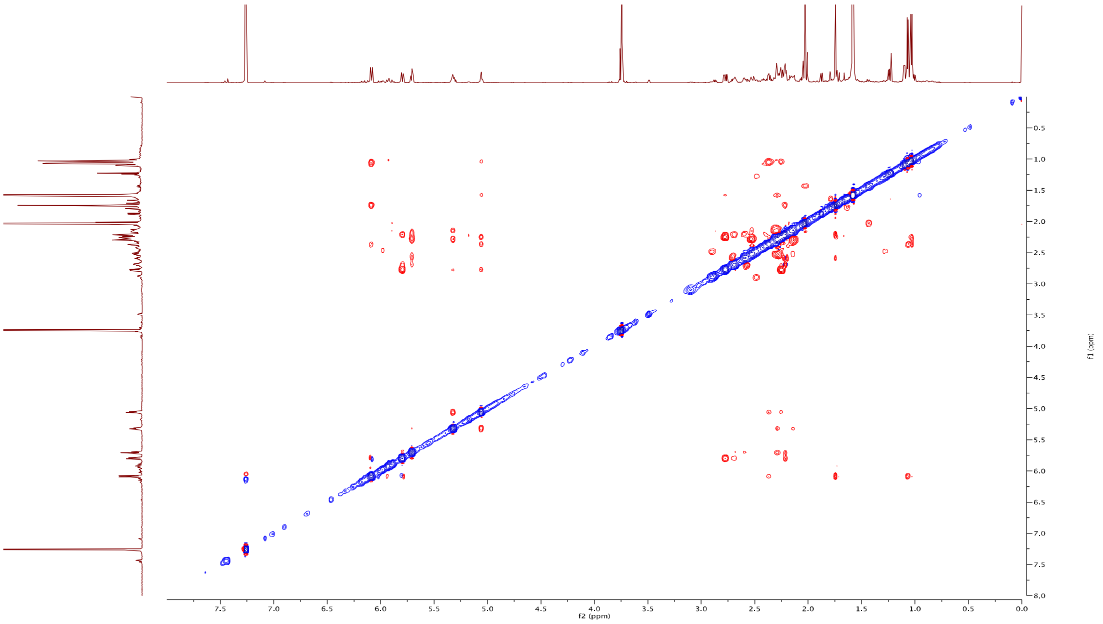

Figure S50. The NOESY spectrum of **6**.

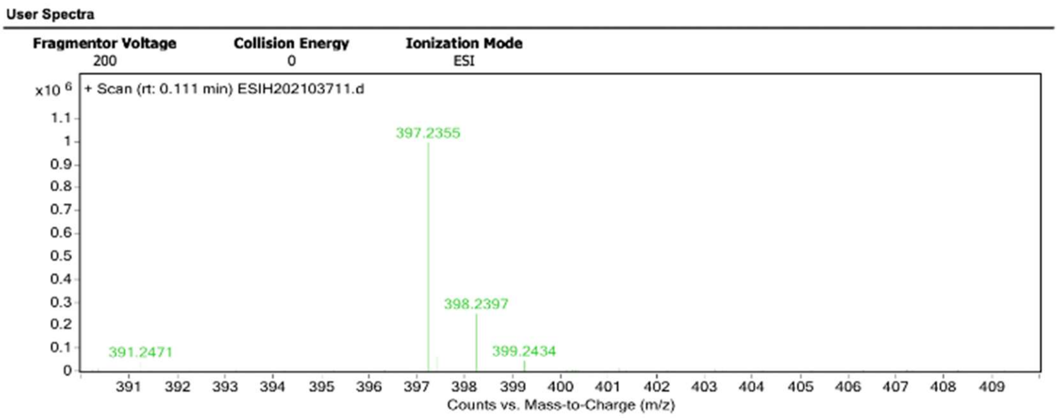

Formula Calculator Results

m/z	Calc m/z	Diff (mDa)	Diff (ppm)	Ion Formula	Ion
397.2355	397.2349	-0.57	-1.43	C23 H34 Na O4	(M+Na)+

--- End Of Report ---

Figure S51. The HR-ESIMS spectrum of **6**.

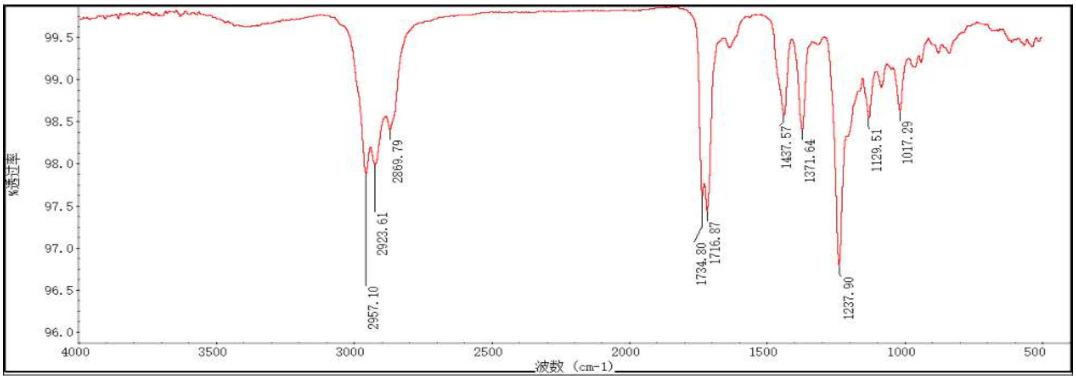

Figure S52. The IR spectrum of **6**.

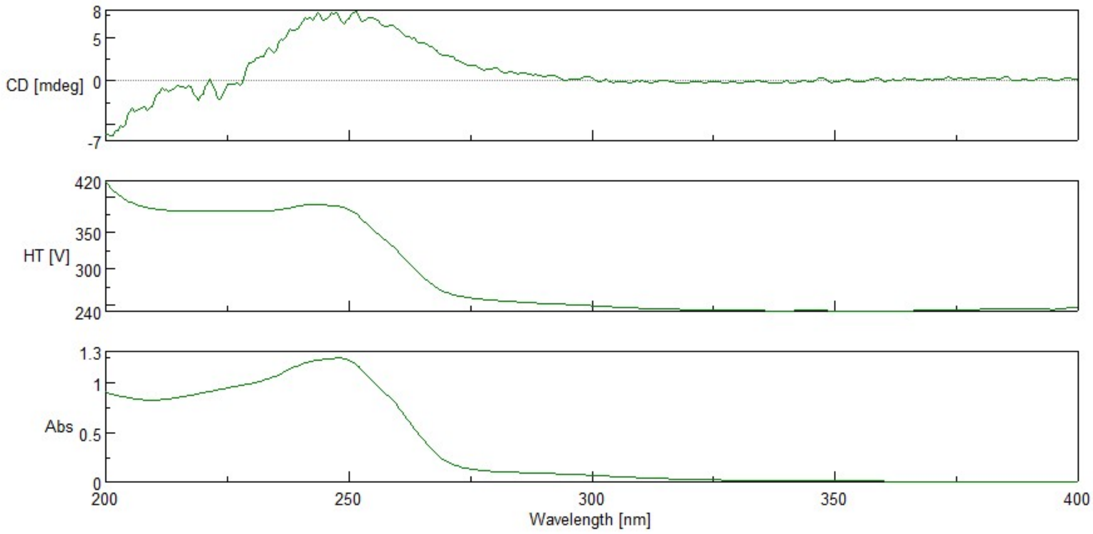

Figure S53. The ECD and UV spectra of **6**.

7. Original spectra of 7

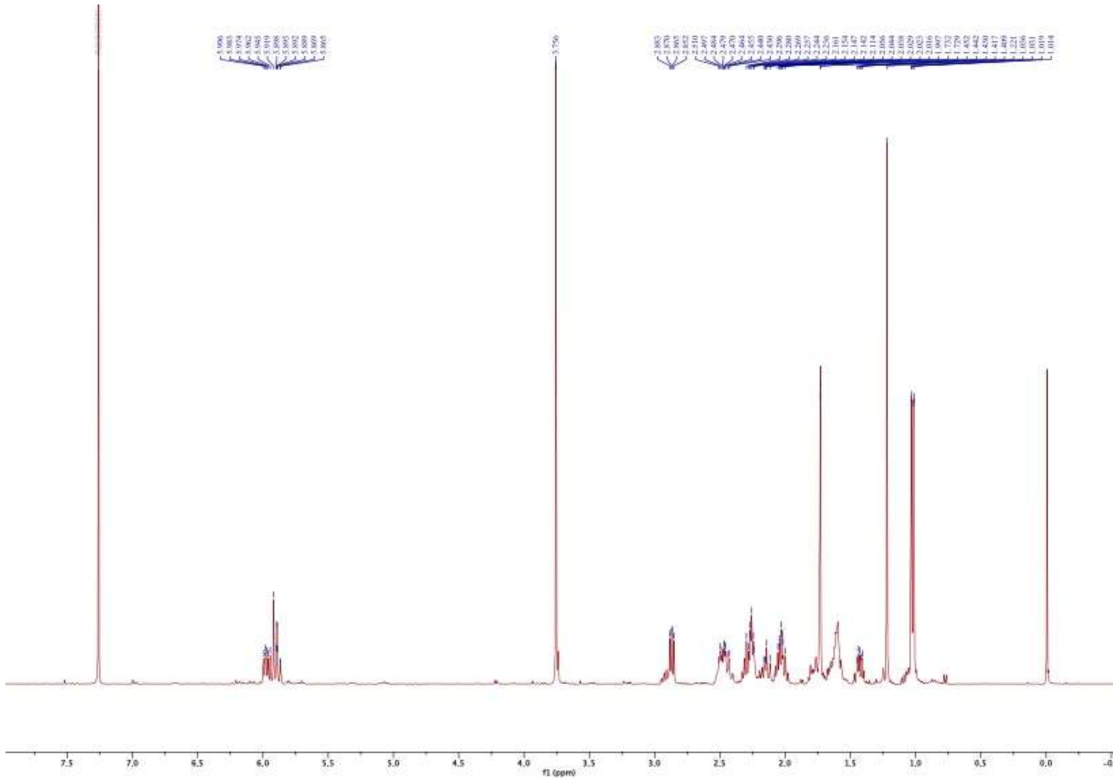

Figure S54. The ^{1}H NMR spectrum of **7**.

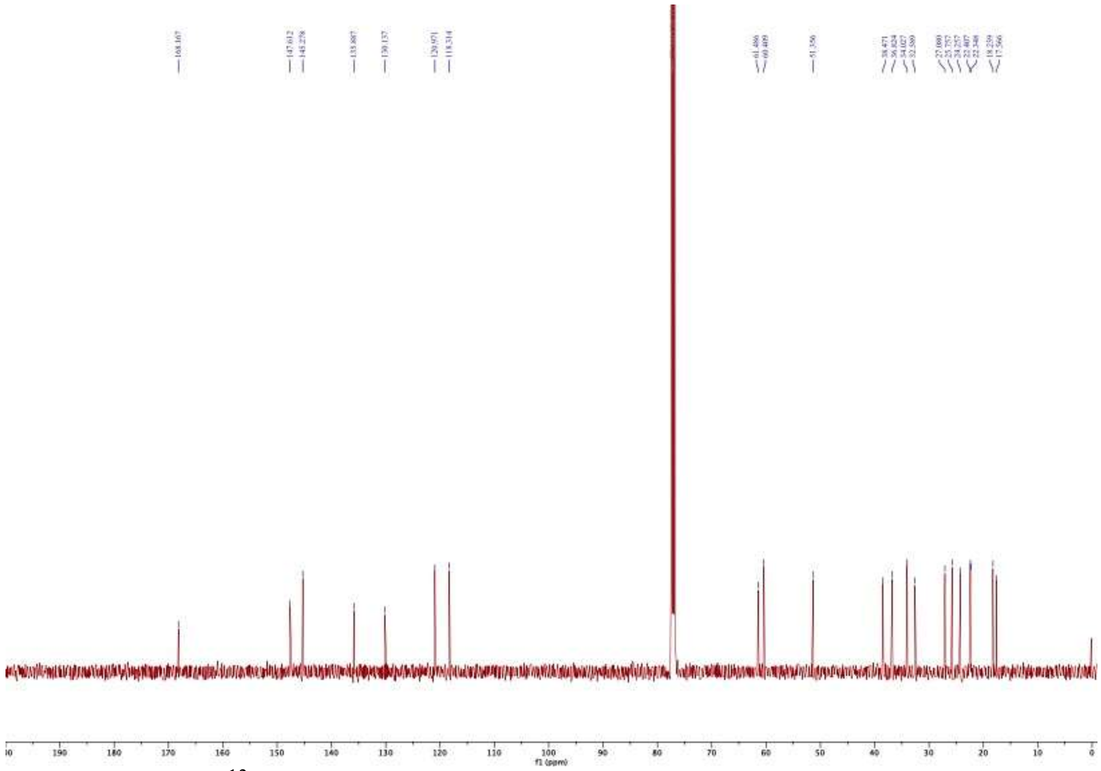

Figure S55. The ^{13}C NMR spectrum of **7**.

8. Original spectra of 8

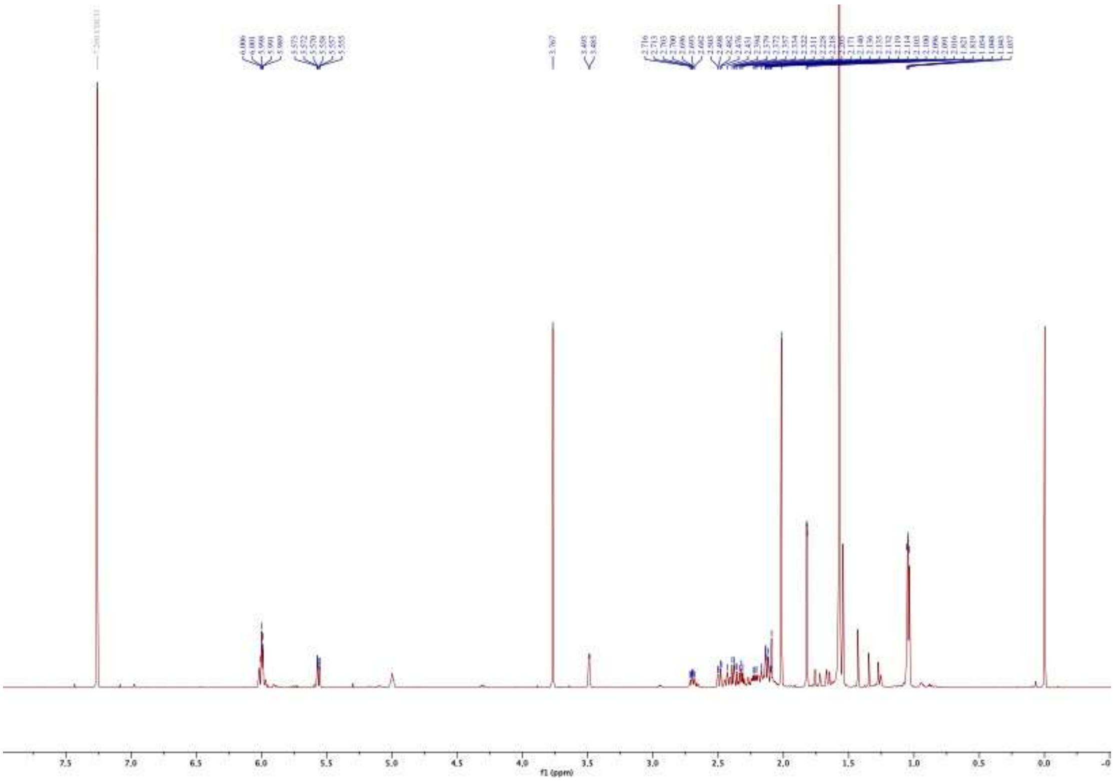

Figure S56. The ^{1}H NMR spectrum of **8**.

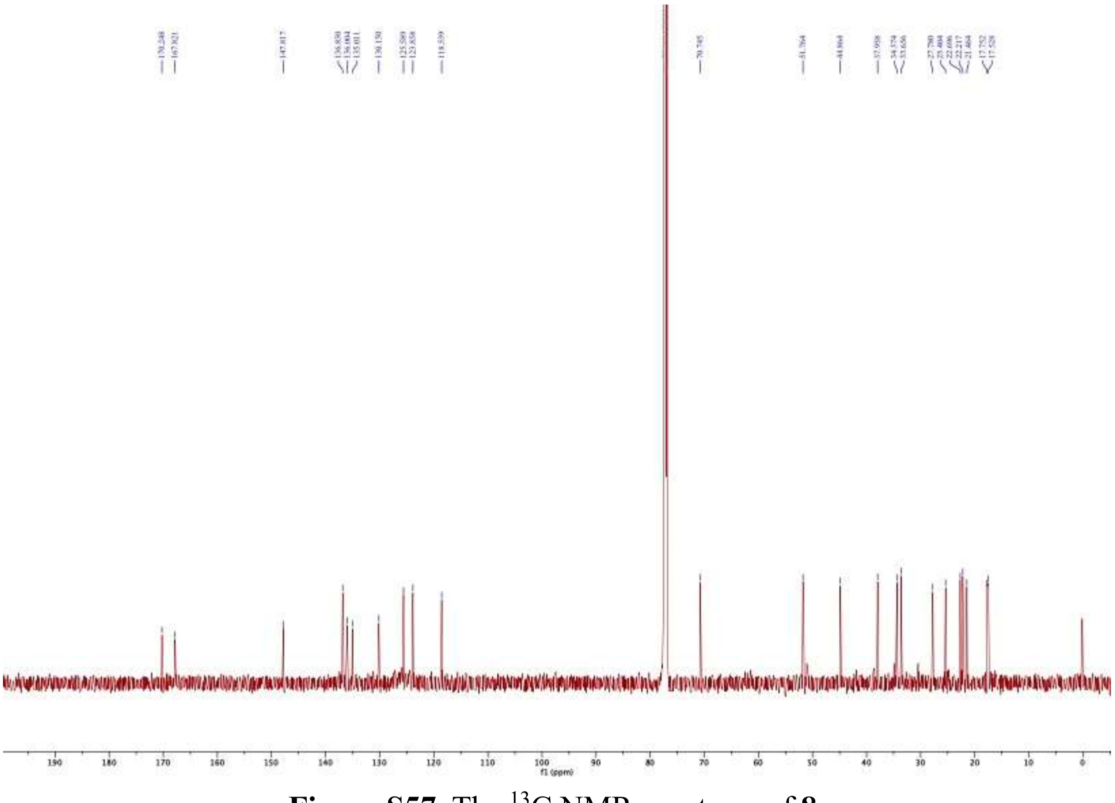

Figure S57. The ^{13}C NMR spectrum of **8**.

9. Original spectra of **9**

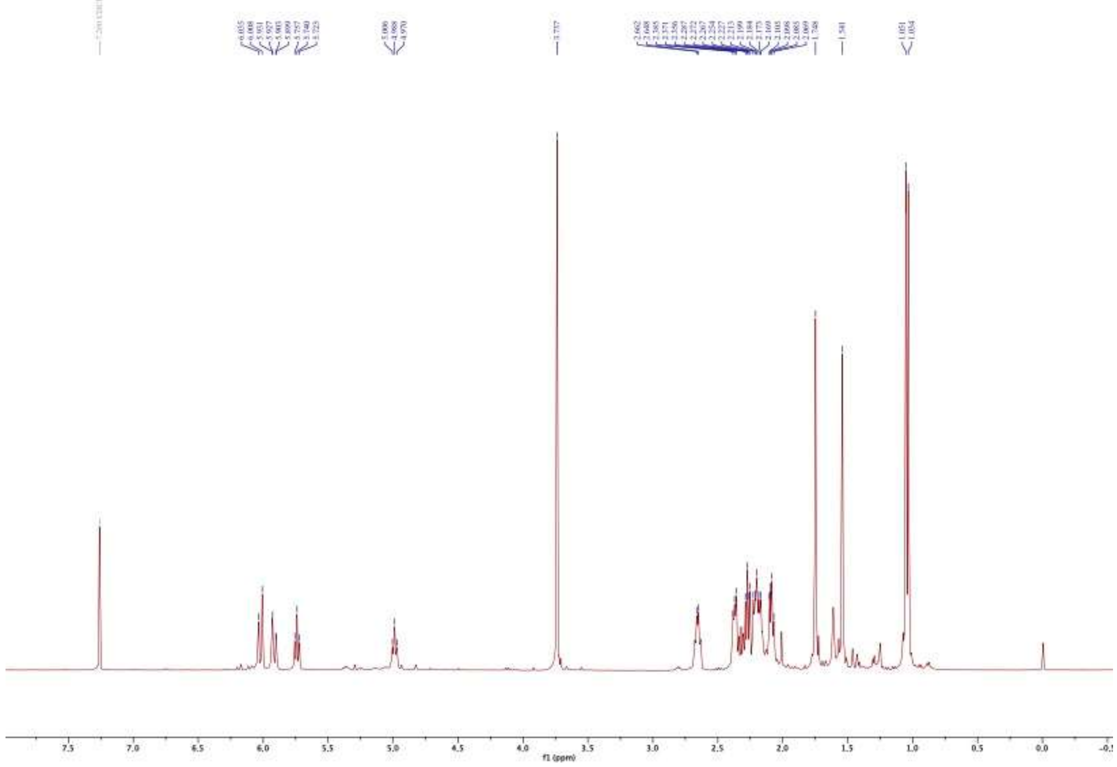

Figure S58. The ^{1}H NMR spectrum of **9**.

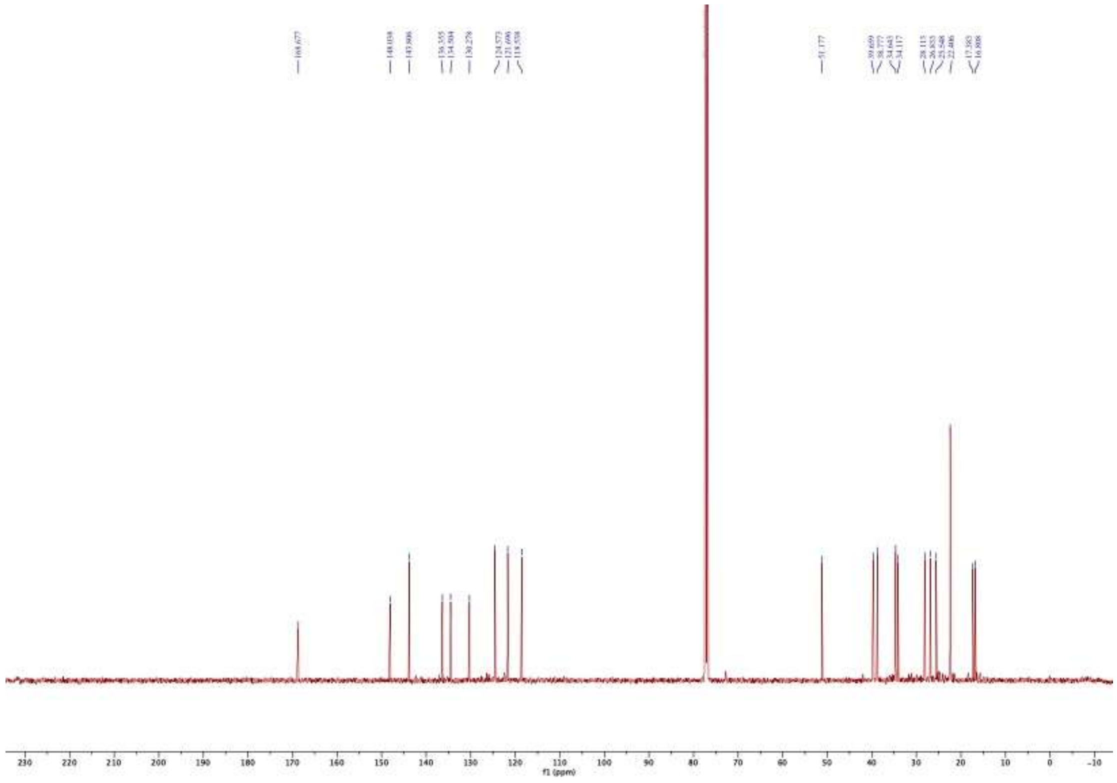

Figure S59. The ^{13}C NMR spectrum of **9**.

10. Acetylation reaction of 5

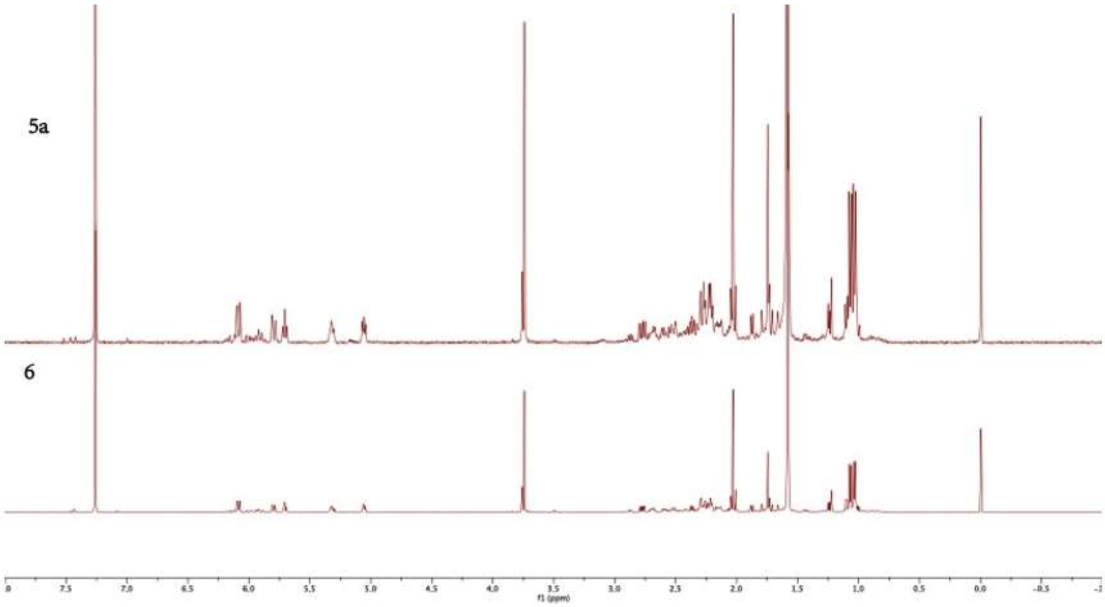

Figure S60. NMR spectra comparation of the acetylated derivative of **5** (**5a**) and **6**.
